# Single-cell mass cytometry reveals complex myeloid cell composition in active lesions of progressive multiple sclerosis

**DOI:** 10.1186/s40478-020-01010-8

**Published:** 2020-08-18

**Authors:** Chotima Böttcher, Marlijn van der Poel, Camila Fernández-Zapata, Stephan Schlickeiser, Julia K. H. Leman, Cheng-Chih Hsiao, Mark R. Mizee, Adelia, Maria C. J. Vincenten, Desiree Kunkel, Inge Huitinga, Jörg Hamann, Josef Priller

**Affiliations:** 1 Department of Neuropsychiatry and Laboratory of Molecular Psychiatry, Charité – Universitätsmedizin Berlin, Berlin, Germany; 2 Neuroimmunology Research Group, Netherlands Institute for Neuroscience, Amsterdam, The Netherlands; 3 BIH Center for Regenerative Therapies (BCRT), Charité – Universitätsmedizin Berlin, Berlin, Germany; 4 Department of Experimental Immunology, Amsterdam University Medical Centers, University of Amsterdam, Amsterdam, The Netherlands; 5 Netherlands Brain Bank, Netherlands Institute for Neuroscience, Amsterdam, The Netherlands; 6 Flow & Mass Cytometry Core Facility, Charité – Universitätsmedizin Berlin and Berlin Institute of Health (BIH), Berlin, Germany; 7 Swammerdam Institute for Life Sciences, Center for Neuroscience, University of Amsterdam, Amsterdam, The Netherlands; 8 German Center for Neurodegenerative Diseases (DZNE), Berlin, Germany; 9 University of Edinburgh and UK Dementia Research Institute (DRI), Edinburgh, UK

**Keywords:** Progressive multiple sclerosis, Mass cytometry, Microglia, Myeloid cells, Active lesion

## Abstract

Myeloid cells contribute to inflammation and demyelination in the early stages of multiple sclerosis (MS), but it is still unclear to what extent these cells are involved in active lesion formation in progressive MS (PMS). Here, we have harnessed the power of single-cell mass cytometry (CyTOF) to compare myeloid cell phenotypes in active lesions of PMS donors with those in normal-appearing white matter from the same donors and control white matter from non-MS donors. CyTOF measurements of a total of 74 targeted proteins revealed a decreased abundance of homeostatic and TNF^hi^ microglia, and an increase in highly phagocytic and activated microglia states in active lesions of PMS donors. Interestingly, in contrast to results obtained from studies of the inflammatory early disease stages of MS, infiltrating monocyte-derived macrophages were scarce in active lesions of PMS, suggesting fundamental differences of myeloid cell composition in advanced stages of PMS.

## Introduction

Multiple sclerosis (MS) is a chronic inflammatory disease of the central nervous system (CNS) which leads to demyelinating lesions and diffuse neurodegeneration spreading throughout the white and grey matter of the brain and the spinal cord [1, 2]. In most cases (85–90% of patients with MS), the disease starts with a relapsing-remitting course (RRMS), which may develop into a progressive course (secondary progressive MS, SPMS) with ongoing neuroinflammation [3, 4]. For some MS patients (10–15% of patients), neurological disability increases progressively over time without relapse or remission (primary progressive MS, PPMS). From a neuropathological perspective, MS lesions are characterized as active, mixed active/inactive, inactive remyelinated (shadow plaques), or inactive lesions, based on demyelination and the presence of HLA-DR^+^ myeloid cells [1, 5]. Both active and mixed active/inactive lesions are characterized by the loss of myelin and the presence of activated foamy microglia/macrophages containing myelin, indicating that microglia/macrophages play a pathogenic role in MS [1, 5]. Active lesions are thought to be the earliest stage in MS lesion formation [5, 6]. As the disease progresses, axonal damage and neurodegeneration become more pronounced. A higher proportion of mixed active/inactive lesions and a less prominent peripheral immune cell infiltrate are observed in progressive MS (PMS) as compared to relapsing disease [5, 7]. Unlike RRMS, immunomodulatory treatments are not effective in patients with PMS [8, 9], suggesting that different pathological processes besides classical neuroinflammation may occur in the progressive form of the disease. As well as B cell-targeted therapies and sphingosine-1-phosphate antagonists, promotion of remyelination and targeting of myeloid cells are promising strategies for treating PMS [10].

Activation of microglia/macrophages is considered a key mechanism which contributes to inflammation, demyelination and neurodegeneration in MS. Using bulk transcriptomic analysis, we have recently demonstrated subtle changes in expression of microglial genes involved in lipid storage and metabolism in normal-appearing white matter (NAWM) in late-stage PMS [11]. These altered microglial signatures are early signs of MS pathology as a similar transcriptional microglial profile was found in chronic active lesions [11]. We have also demonstrated overall preservation of microglial homeostatic functions in NAWM PMS tissue [11]. However, the phenotypic heterogeneity of microglia regarding their homeostatic and inflammatory state in PMS active lesions remains unknown. A landmark study using single-cell RNA-sequencing (scRNA-Seq) showed unique transcriptomic profiles of microglia in active lesion biopsies from patients in the early disease stages of MS, compared with microglia isolated from control tissue of non-MS donors [12]. Further, in the early disease stages of MS and in a mouse model of demyelination, homeostatic microglial genes such as *P2RY12*, *TMEM119* and *CX3CR1* were downregulated in active lesions, whereas genes associated with microglia states *SPP1*, *CD74* and *CTSD* and the cytokine *CCL4* were upregulated [12, 13]. However, it is yet to be investigated whether these changes can also be detected in active lesions of PMS at the single-cell protein level. Furthermore, whereas approximately 10% of Iba1^+^ cells in brain sections of patients with early MS are infiltrating monocytes [12], it is not yet known whether a similar contribution of monocyte-derived cells to MS lesion initiation and/or maturation can be detected in active lesions of PMS. Together, microglia show context-dependent signatures in lesions of early MS, but the differential functions of microglia and the involvement of infiltrating monocyte-derived macrophages in PMS are not clear.

In this study, we have used single-cell mass cytometry by time of flight (CyTOF) to comprehensively characterize the phenotypes of myeloid cells in active lesions and in NAWM from ten PMS donors. Subsequently, we compared these cells to those isolated from control WM of eight non-MS donors. The results obtained from this study suggest that active lesions of PMS contain diverse clusters of highly phagocytic and activated WM myeloid cells with little infiltration of monocyte-derived macrophages.

## Materials and methods

### Human post-mortem tissue

Post-mortem tissue of brain donors was provided by the Netherlands Brain Bank (NBB, Amsterdam, The Netherlands, www.brainbank.nl). All brain donors gave informed consent to perform autopsies and to use tissue, clinical and neuropathological information for research purposes, approved by the Ethics Committee of VU medical center (Amsterdam, The Netherlands).

Subcortical white matter (WM) tissue was collected from non-MS WM control donors (*n* = 8), and from MS donors we collected subcortical NAWM (*n* = 10) and subcortical WM lesions (n = 10). NAWM MS tissue was dissected on post-mortem magnetic resonance imaging (MRI) guidance during autopsy [14]. In addition, macroscopically visible MS lesions were dissected by a neuropathologist.

Neurological diagnoses were confirmed by a neuropathologist. Information on MS diagnosis and disease duration was obtained from clinical data, showing that all donors were diagnosed with progressive MS, 3 donors with primary progressive MS and 7 donors with secondary progressive MS. Donor characteristics and post-mortem variables are displayed in Additional file 1, Supplementary Table 1-3 and 6.

### MS lesion characterization

From post-mortem tissue that was taken out for microglia isolation, a small part was snap-frozen in liquid nitrogen and stored in − 80 °C until further use. Frozen tissue sections (20 μm) of control WM, NAWM and MS lesions were cut and dried overnight. For immunohistochemistry, these sections were fixed for 15 min with 4% paraformaldehyde in phosphate buffered saline (PBS) pH 7.6, followed by endogenous peroxidase blocking in 1% H_2_O_2_ in PBS for 20 min. Sections were incubated with primary antibodies HLA-DR/DQ/DP (1:1000, M0775; Dako, Glostrup, Denmark) or PLP (1:3000, MCA839G; Serotec, Oxford, UK) in incubation buffer (0.5% Triton X-100 and 1% bovine serum albumin (BSA) in PBS) overnight at 4 °C. Secondary antibodies were incubated for 1 h at room temperature (RT); for HLA-DR biotinylated anti-mouse (1:400, BA-2001; Vector Laboratories, Burlingame, CA, USA) was diluted in incubation buffer, for PLP the HRP-labeled mouse antibody (K5007, Dako Real EnVision detection system; Dako) was used. Next, sections for HLA-DR staining were incubated for 45 min in avidin-biotin complex (1:800, PK-6100; Vector Laboratories) at RT, followed by 3,3′-diaminobenzidine (DAB) incubation (1:100, K5007; Dako) for 10 min at RT, for both HLA-DR and PLP stainings. Immunoreactivity was examined using an Axioskop980 microscope (Zeiss, Oberkochen, Germany) and Photomacroscope M420 (Wild Heerbrugg, Zwitserland) to characterize lesions based on HLA-DR presence and morphology of HLA-DR^+^ cells together with myelin intactness based on PLP staining [5].

### Microglia isolation

Microglia were isolated from post-mortem WM tissue, as described previously [11, 15]. Briefly, post-mortem tissue that was collected during autopsy was stored in Hibernate-A medium (Invitrogen, Carlsbad, CA, USA) at 4 °C until further processing. Within 24 h, the tissue was homogenized for 5 min in Hibernate-A medium supplemented with DNAseI (10 mg/ml; Roche, Basel, Switzerland), using a tissue homogenizer (VWR, Radnor, PA, USA). Next, undiluted Percoll (density of 1.13 g/ml; GE Healthcare, Little Chalfont, UK) was added to form a single gradient for density centrifugation and the interlayer was collected for magnetic activated cell sorting (MACS; Miltenyi, Bergisch Gladbach, Germany) using CD11b magnetic beads (catalogue number #130–049-601, Miltenyi Biotech). Viable cells were counted using a hemocytometer (Optic Labor, Friedrichshof, Germany) or eFluor™ 506. Cells were then collected in beads buffer (0.5% BSA + 2 mM EDTA in PBS, pH 7.6) for flow cytometry analysis. Using this protocol, about 95% of viable cells were identified as myeloid cells (Supplementary Fig. 1). For CyTOF analysis, CD11b^+^ cells were incubated for 11 min in fixation/stabilization buffer (Smart Tube Inc., San Carlos, CA, USA) and stored in − 80 °C.

### IRF8^+^ nuclei isolation and sorting

IRF8^+^ nuclei were isolated and sorted as described previously [11]. Briefly, frozen tissue from MS donors, NAWM tissue (*n* = 7) and tissue containing MS lesions (*n* = 5), matched for age, was provided by the NBB. For each tissue block, the first and last section were double stained for HLA-DR/PLP to determine microglia activation and myelin integrity. MS lesions were characterized as previously described by Luchetti and colleagues [5].

From each tissue block, 10–12 sections of 50 μm thickness were cut and homogenized in 1 ml homogenization buffer (1 μm DTT (Thermo Fischer Scientific), 1x protease inhibitor (Roche), 80 U/ml RNAseIN (Promega, Madison, WI, USA) and 1% Triton X-100) with nuclei isolation medium #1 (NIM #1; 250 mM sucrose, 25 mM KCL, 5 mM MgCl_2_, 10 mM Tris buffer pH 8 diluted in nuclease free water) filtered through a 30-μm cell strainer. The amount of nuclei was counted using a hemocytometer (Optic Labor) and nuclei were incubated with Hoechst (#H3570, 1:1000; Invitrogen) and IRF8 antibody (#566373, PE-labeled, 1:50, clone U31–644; BD Biosciences, San Diego, CA, USA) in staining buffer (0.5% RNAse free BSA, 1% normal human serum and 0.2 U/μl RNAseIn in RNAse-free PBS, pH 7.4) for 1 h at 4 °C. Isotype control antibody IgG-PE (#12–4714-42, clone P3.6.8.1, 1:25; Invitrogen) was used to determine background staining.

Stained nuclei were sorted using a Sony SH800S cell sorter (Sony Biotechnology, San Jose, CA, USA). The Hoechst and IRF8 double positive nuclei fractions was collected and lysed in RNA lysis buffer (RNeasy Isolation mini kit; Qiagen, Hilden, Germany).

### RNA isolation

RNA from sorted IRF8^+^ nuclei was isolated using the RNeasy Mini kit (Qiagen), according to the manufacturer’s protocol. Lysed samples were mixed with 70% ethanol and transferred to a mini spin column. After washing steps, elution was collected in 20 μl deionized water.

### DNA synthesis and quantitative real-time PCR

The Quantitect Reverse Transcription Kit (Qiagen) was used for cDNA synthesis. According to manufacturer’s protocol, isolated RNA (25 ng) from sorted IRF8^+^ nuclei was mixed with gDNA wipeout buffer, incubated for 2 min at 42 °C and put on ice. Next, Quantiscript RT buffer, RT primer mix and Quantiscript Reverse Transcriptase were mixed and incubated with RNA sample at 42 °C for 30 min, followed by 3 min incubation at 95 °C.

For RT-qPCR, 0.6 ng cDNA was mixed with 17 μl SYBR Green PCR master mix (Applied Biosystems, Foster City, CA, USA) and 2 μl primer pairs. Samples were measured and analyzed using 7300 RT-PCR machine and software (Applied Biosystems).

Primer pairs were designed at the Integrated DNA Technologies website (eu.ifdna.com), using the PrimerQuest tool. For primer design the following criteria were used: same Tm, 50% GC content, amplicon size between 80 and 140 base pairs and exclude primers that span introns, to detect unspliced nuclear DNA. Primer pairs were checked for specificity using cDNA derived from pooled MS and control donor brain tissue. Optimal primers (Additional file 1: Supplementary Table 4) were selected based on dissociation curve, and 8% sodium dodecyl sulfate polyacrylamide gel electrophoresis gel was used to detect PCR product and exclude primer pairs that can form dimers. Gene expression was normalized to the mean of 2 housekeeping genes, glyceraldehyde 3-phosphate dehydrogenase (*GAPDH*) and elongation factor-1 alpha (*EEF1A1*). Target gene expression values were calculated using the 2- ΔΔCT method.

### Flow cytometric analysis

Isolated microglia from MS donors (*n* = 7) were incubated for 15 min in FcR-blocking buffer (1:5; Miltenyi Biotec), to block unspecific binding of antibodies to Fc-receptors. Next, microglia were incubated with conjugated primary antibodies (Additional file 1: Supplementary Table 5) diluted in beads buffer (0.5% BSA and 2 mM EDTA in PBS, pH 7.6) for 30 min at 4 °C. To determine viability, cells were incubated with viability dye efluor506 (Additional file 1: Supplementary Table 5).

To assess minimal phenotyping of isolated microglia, CD45 and CD11b expression was determined. In addition, expression of homeostatic microglia receptors, P2Y_12_, CX3CR1 and GPR56 was measured. To exclude infiltrating leukocytes in the samples collected from MS lesion tissue, CD3, CD19, CD56 and CD66b were included.

Surface protein expression was detected on a 3-laser BD FACSCanto II machine (BD Biosciences) with software BD DIVA version 8.1. FlowJo software version 10.1 (Ashland, OR, USA) was used to determine median fluorescence intensity.

### Immunohistochemical quantification

Paraffin tissue blocks from age-matched control (*n* = 5) and MS (*n* = 11) donors (Additional file 1: Supplementary Tables 1 and 6) were cut into 8 μm-thick sections. Tissue sections were deparaffinized with xylene and rehydrated in ethanol series, followed by antigen retrieval with citrate buffer pH 6 for 20 min in a steamer. Sections were blocked in 10% normal horse serum/normal donkey serum for 30 min and incubated with P2Y_12_ antibody and either CD68 (DAKO, # M0814) or HLA-DR (DAKO, #M0775) antibodies diluted in incubation buffer (0.5% Triton-X100 and 0.25% gelatin in tris-buffered saline (TBS, pH 7.6) and incubated overnight at 4 °C. After overnight incubation with primary antibody, samples were incubated for 2 h at RT with Alexa Fluor 568 and Alexa Fluor 488-conjugated secondary antibody. Nuclei were stained with DAPI. All images were acquired in a Leica TCS SP5 microscope (Leica microsystems). P2Y_12_^+^DAPI^+^ and P2Y_12_^+^CD68^+^DAPI^+^ or P2Y_12_^+^HLA-DR^+^DAPI^+^ cells were counted using IMARIS software. All image processing for visualization was performed with ImageJ software.

### Intracellular barcoding for mass cytometry

Percoll-isolated myeloid cells were fixed with fixation/stabilization buffer (SmartTube) [16] and frozen at − 80 °C until analysis by mass cytometry. Cell were thawed and subsequently stained with premade combinations of six different palladium isotopes: ^102^Pd, ^104^Pd, ^105^Pd, ^106^Pd, ^108^Pd and ^110^Pd (Cell-ID 20-plex Pd Barcoding Kit, Fluidigm). This multiplexing kit applies a 6-choose-3 barcoding scheme that results in 20 different combinations of three Pd isotopes. After 30 min staining (at room temperature), individual samples were washed twice with cell staining buffer (0.5% bovine serum albumin in PBS, containing 2 mM EDTA). All samples were pooled together, washed and further stained with antibodies.

### Antibodies

Anti-human antibodies (Additional file 1: Supplementary Tables 7 and 8) were purchased either pre-conjugated to metal isotopes (Fluidigm) or from commercial suppliers in purified form and conjugated in house using the MaxPar X8 kit (Fluidigm) according to the manufacturer’s protocol. Using different cell types from different body compartments, each antibody was titrated and validated as into the working panels prior to use to ensure that the resulted signals were informative [16, 17].

### Cell-surface and intracellular staining

After cell barcoding, washing and pelleting, the combined samples were stained and processed as described previously [16, 17]. Briefly, cells were re-suspended in 100 μl of antibody cocktail directed against cell surface markers (Additional file 1: Supplementary Tables 7 and 8) and incubated for 30 min at 4 °C. Then, cells were washed twice with cell staining buffer (PBS containing 0.5% BSA and 2 mM EDTA). For intracellular staining, the stained (non-stimulated) cells were then incubated in fixation/permeabilization buffer (Fix/Perm Buffer, eBioscience) for 60 min at 4 °C. Cells were then wash twice with permeabilization buffer (eBioscience). The samples were then stained with antibody cocktails directed against intracellular molecules (Additional file 1: Supplementary Tables 7 and 8) in permeabilization buffer for 1 h at 4 °C. Cells were subsequently washed twice with permeabilization buffer and incubated overnight in 4% methanol-free formaldehyde solution. The fixed cells were then washed and re-suspended in 1 ml iridium intercalator solution (Fluidigm) for 1 h at RT, followed by two washes with cell staining buffer and two washes with ddH_2_O (Fluidigm). Finally, cells were pelleted and kept at 4 °C until CyTOF measurement.

### Bead staining

For the bead-based compensation of the signal spillover, AbC total antibody compensation beads (Thermo Fisher Scientific) were single stained with each of the antibodies used in all three antibody panels according to manufacturer’s instructions. Stained beads were then measured with CyTOF and the compensation matrix was then generated [17, 18].

### CyTOF measurement

Cells were analysed using a CyTOF2 upgraded to Helios specifications, with software version 6.7.1014 [16, 17], using a narrow bore injector. The instrument was tuned according to the manufacturer’s instructions with tuning solution (Fluidigm) and measurement of EQ four element calibration beads (Fluidigm) containing ^140/142^Ce, ^151/153^Eu, ^165^Ho and ^175/176^Lu served as a quality control for sensitivity and recovery.

Directly prior to analysis cells were re-suspended in ddH_2_O, filtered through a 20-μm cell strainer (Celltrics, Sysmex), counted and adjusted to 5–8 × 10^5^ cells/ml. EQ four element calibration beads were added at a final concentration of 1:10 v/v of the sample volume to be able to normalize the data to compensate for signal drift and day-to-day changes in instrument sensitivity.

Samples were acquired with a flow rate of 300–400 events/s. The lower convolution threshold was set to 400, with noise reduction mode turned on and cell definition parameters set at event duration of 10–150 pushes (push = 13 μs). The resulting flow cytometry standard (FCS) files were normalized and randomized using the CyTOF software’s internal FCS-Processing module on the non-randomized (‘original’) data. The default settings in the software were used with time interval normalization (100 s/minimum of 50 beads) and passport version 2. Intervals with less than 50 beads per 100 s were excluded from the resulting FCS file.

### Mass cytometry data processing and analysis

Following the workflow from our previous study [16, 17], Cytobank (www.cytobank.org) was used for initial manual gating on live single cells and Boolean gating for de-barcoding. Nucleated single intact cells were manually gated according to DNA intercalators ^191^Ir/^193^Ir signals and event length. For de-barcoding, Boolean gating was used to deconvolute individual sample according to the barcode combination. Prior to data analysis, each FCS file was compensated for signal spillover using R package *CATALYST* [18]. For dimensionality reduction, visualization and further exploration, (2D) tSNE maps were generated according to the expression levels of all markers in each panel. For embedding, we set hyperparameters to perplexity of 30, theta of 0.5, and iterations of 1000 per 100,000 analysed cells. To visualize marker expression arcsinh transformation was applied to the data. All FCS files were then loaded into R and further data analysis was performed with an in-house written script based on the workflow proposed by M. Nowicka and colleages [19]. Briefly, for unsupervised cell population identification we performed cell clustering with the *FlowSOM* [20] and *ConsensusClusterPlus* [21] packages using all markers (*Exp-I*) or TYPE markers (*Exp-II* and *-III*). We then performed visual inspection of cluster-coloured tSNE plots and phenotypic heatmaps for a more detailed profile of each cluster and determined the number of meta-clusters on the basis of delta area under cumulative distribution function (CDF) curve and k value of the clustering analysis and the consistency of phenotypes for statistical test. For detection of differential abundance of clusters between conditions we used generalized linear mixed models (GLMM) performed with the *diffcyt* package [17], with a false discovery rate (FDR) adjustment (Benjamini-Hochberg (BH) procedure) for multiple hypothesis testing. A *P* value < 0.05 (unadjusted) and < 0.05 (FDR-BH adjusted) was considered statistically significant.

### Imaging mass cytometry

Paraffin tissue microarray (TMA) blocks containing samples from control, NAWM and lesion were cut into 5 μm-thick sections. Sections were deparaffinized with xylene and rehydrated in ethanol series, followed by heat-induced antigen retrieval in Tris-EDTA buffer (pH = 9.0) for 20 min at 95 °C in a steamer. The sections were then blocked with 3% purified BSA in 0.1% Triton-X PBS for 1 h at RT. Sections were incubated overnight at 4 °C with anti-P2Y_12_ conjugated with biotin. After washing, all sections were incubated with metal-conjugated antibodies (Additional file 1: Supplementary Table 9) overnight at 4 °C. Nuclei were detected using an Ir-Intercalator (1:500). Samples were then dried and stored at RT until measurement.

### Imaging mass cytometry acquisition and data analysis

Imaging mass cytometry was performed on a CyTOF2/upgraded to Helios specifications coupled to a Hyperion Tissue Imager (Fluidigm), using CyTOF software version 6.7.1014. Prior to ablation the instrument was tuned according to the manufactures instructions, using the 3-Element Full Coverage Tuning Slide (Fluidigm). The dried slide was loaded into the imaging module and regions of interest were selected for each sample of the TMA on a preview (panorama). Optimal laser power was determined for each sample to obtain complete ablation of the tissue. Laser ablation was performed at a resolution of 1 μm and a frequency of 200 Hz. Data were stored as MCD files as well as txt files. Original files were opened with MCD viewer and single 16-bit images were extracted as. TIFF files. For visualization only, images were transferred to ImageJ and the different channels were merged. A Gaussian blurr (kernel width, 0.70 pixels) was used for noise reduction.

For single-cell analysis, we first processed images from each sample using *Ilastik* [22], an open-source program that uses interactive machine learning to separate single cells from background. The program was trained to identify DNA iridium-intercalator as nuclei and P2Y_12_-^Ho^165 as cell membrane, and the pixel classificator was then applied to all images. As a result, a binary mask delimiting each single-cell was obtained and transferred on to *CellProfiler* [23]. We applied a set of modules to create single-cell masks, the modules included filters for cell size, negative selection for cells on the border of the image or exclusion of cytoplasm signal with no nuclei, thus generating 16-bit *.tiff* single-cell masks with only full cells for each image. Each of the *.tiff* files and single-cell masks were then transferred to *histoCAT* [24] for further analysis. In *histoCAT*, we ran a dimensionality reduction tSNE algorithm to visualize single cell data from all samples. We then ran a Phenograph analysis in which cells were clustered according to their marker expression (for markers CD11c, CD44, CD45, CD68, HLA-DR, P2Y_12_ and TNF, using k = 50 nearest neighbours). The mean expression and cell frequencies per sample/cluster where then extracted using R.

### Statistical analysis

No randomization and blinding strategies were applied in this study. However, data processing and analysis, as well as statistical testing were carried out in an unsupervised manner. No priori statistical methods were used to predetermine sample sizes due to sample accessibility and insufficient previous data to enable this. However, sample sizes were chosen based on estimates of anticipated variability through previous studies on scRNA-Seq analysis of microglia in acute lesion MS [12]. Dichotomous variables of the sample cohort were analysed with Fisher’s exact test (GraphPad Prism). Quantitative data are shown as independent data points with median or Box-Whisker. Unless otherwise stated, analyses of statistical significance were performed by computational analysis using generalized linear mixed-effects model (GLMM) available through R package *diffcyt* and false discovery rate (FDR) adjustment (using Benjamini-Hochberg procedure) for multiple hypothesis testing. A *p*-value < 0.05 (FDR-adjusted) was considered statistically significant.

## Results

### Characterization of MS lesions

This study used post-mortem WM brain from ten MS donors. All donors were diagnosed with PMS and had a mean disease duration of 24.7 years (*sd* = 11 years), and an average disease severity (defined as years until the patients reached an expanded disability status scale (EDSS) score of 6.0) of 13.1 years (*sd* = 7.8) (Additional file 1: Supplementary Tables 1 and 2).

For each MS donor, the NAWM tissue was dissected using magnetic resonance imaging (MRI) guidance [14] and MS lesions were dissected by a neuropathologist based on macroscopic appearance. Active MS lesions were characterized as previously described [5, 25]. For each MS donor, myeloid cells were isolated from a block of NAWM tissue and a block of active lesion tissue surrounded by NAWM, using an optimized protocol involving density gradient separation and CD11b-magnetic bead sorting (MACS) [15]. To confirm the active status of MS lesions, immunohistochemical analysis was retrospectively performed on tissue blocks. Myelin proteolipid protein (PLP) and human leukocyte antigen (HLA)-DR were used to define myelin integrity and microglia/macrophage activation and morphology, respectively (Additional file 2: Supplementary Fig. 2a). PLP staining of NAWM tissue sections showed intact myelin (Additional file 2: Supplementary Fig. 2a). In contrast, loss of PLP expression, which indicates demyelination, was used to identify active lesions (Additional file 2: Supplementary Fig. 2a). HLA-DR positive cells were present throughout the entire lesion and the majority of microglia/macrophages in active lesions had amoeboid or foamy morphology (Additional file 2: Supplementary Fig. 2a). However, using bulk quantitative polymerase chain reaction (qPCR) analysis of isolated IRF8^+^ nuclei (comprising microglia and macrophages) from frozen tissue sections [11] (Supplementary Table 3), we could not detect significant alterations of heteronuclear RNA expression of homeostatic genes *CX3CR1, TMEM119, P2RY12* and *ADGRG1* in active lesions of PMS, compared to NAWM (Additional file 1: Supplementary Table 4; Additional file 2: Supplementary Fig. 2b). Low-dimensional flow cytometric analysis also revealed no significant differences in the expression levels of the microglial homeostatic proteins CX3CR1, P2Y_12_ and GPR56 (*ADGRG1*) in active MS lesions compared to NAWM (Additional file 1: Supplementary Table 5; Additional file 2: Supplementary Fig. 2c). However, immunohistochemical analysis of the tissue revealed a reduction of P2Y_12_-expressing cells in active lesions of PMS as compared to NAWM from MS donors and control WM tissues from non-MS donors (Additional file 1: Supplementary Table 1 and 6; Additional file 2: Supplementary Fig. 2d). No significant difference in the number of P2Y_12_-expressing cells was found between NAWM and non-MS white matter. Increased expression of HLA-DR was found in P2Y_12_^+^ cells in active lesions, compared to those in NAWM (Supplementary Fig. 3). Slightly enhanced expression of CD68 was also detected in P2Y_12_^+^ cells in active lesions but was not statistically significant (Supplementary Fig. 3).

### Majority of active lesion microglia in PMS preserve homeostatic signatures

To prove an assumption that subtle changes of microglia (which may have been obscured in bulk analysis and/or in low-dimensional phenotypic profiling) characterize active lesions of PMS, we next investigated microglia/macrophage phenotypes in PMS at single-cell resolution. Three multiplexed single-cell CyTOF analyses were performed on MACS-sorted CD11b-expressing cells from active lesions and NAWM (from ten PMS donors), using three different antibody panels (*Exp-I*, *−II* and *-III*; Additional file 1: Supplementary Table 8). With this experimental design, we aimed to demonstrate the reproducibility of the obtained results, along with in-depth phenotypic profiling using a total of 74 antibodies.

**Fig. 1:**
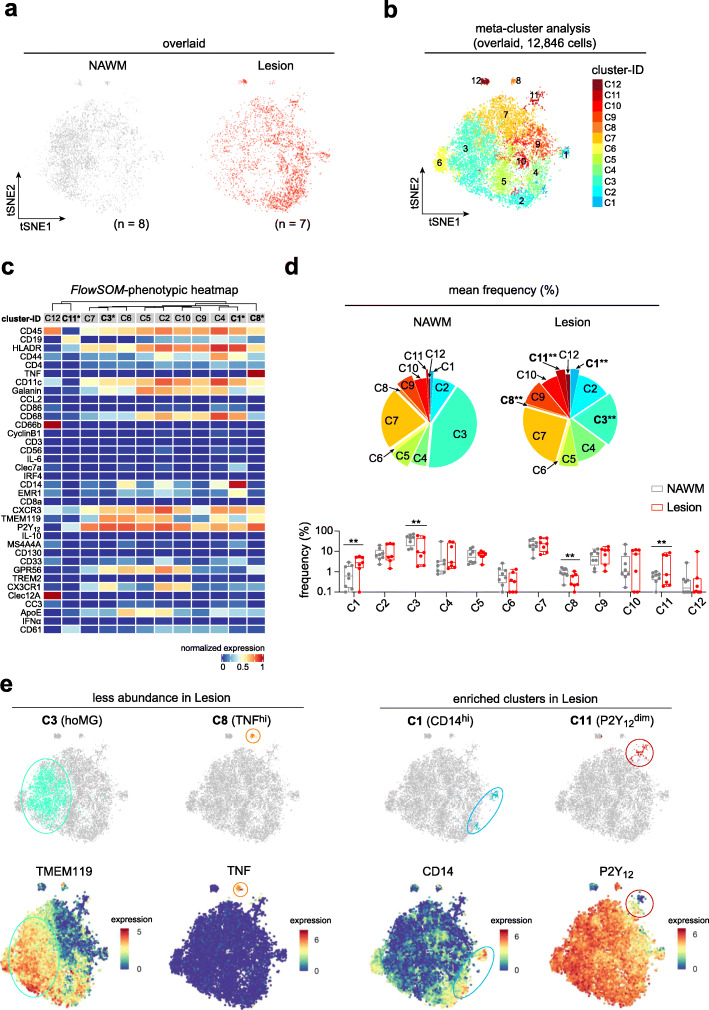
Comparative phenotypic analysis of the CNS myeloid cells in NAWM and active lesions of PMS. **a** The overlaid t-SNE plots of 8 NAWM (grey dot) and 7 active lesions (red dot). The 2D t-SNE maps were generated based on expression levels of all markers of *Exp-I* (Supplementary Table 8). **b** The overlaid t-SNE plot of all samples. The coloring indicates 12 clusters representing diverse myeloid cell phenotypes, defined by the *FlowSOM* algorithm. **c** Heat map cluster demonstrates the expression levels of all 36 markers used for the cluster analysis. Asterisk indicates differentially abundant clusters. Heat colors of expression levels have been scaled for each marker individually (to the 1st and 5th quintiles) (red, high expression; blue, low expression). **d** Pie charts showing the proportions of 12 defined clusters in the two groups. Four differentially abundant clusters (**C1, C3, C8,** and **C11**) between active lesions and NAWM were found. An FDR-adjusted *p* value < 0.05 was considered statistically significant, determined using GLMM (*p < 0.05; **p < 0.01, adjusted). Box-plots show frequencies (%) of all defined clusters. Boxes extend from the 25th to 75th percentiles. Whisker plots show the min (smallest) and max (largest) values. The line in the box denotes the median. Each dot represents the value of each sample. **e** Reduced-dimensional single-cell t-SNE maps highlight all four differentially abundant clusters. In the lower panel, t-SNE maps show the expression of TMEM119, TNF, CD14 and P2Y_12_. Color spectrum indicates expression levels of the marker (red, high expression; blue, low expression)

In *Exp-I*, the antibody panel (Additional file 1: Supplementary Table 8) was designed to characterize microglia as well as to detect the major circulating immune cell subsets including myeloid cells, T, B and natural killer (NK) cells using 36 antibodies recognizing CX3CR1, P2Y_12_, TMEM119, GPR56, TREM2, EMR1, ApoE, Clec7A, MS4A4A, CC3, CD45, CD44, CD19, CD3, CD4, CD8a, CD56, CD66b, CD14, IRF4, Clec12A, HLA-DR, CD11c, CD130, CD86, CD33, CXCR3, Galanin, CD61, CD68, IL-10, IL-6, CCL2, IFN-α, TNF and cyclinB1. First, we embedded all cells from NAWM (*n* = 8) and active lesion WM tissue (*n* = 7) on a reduced dimension t-SNE map (Fig. 1a; Additional file 2: Supplementary Fig. 4). To identify differentially abundant rare cell populations or different cell states between conditions, we performed an exploratory meta-clustering using the FlowSOM algorithm (*FlowSOM/Consensus-ClusterPlus*) [18–21]. Importantly, the number of clusters defined may not necessarily represent functionally distinct subsets of myeloid cells, as it could also include transient cell states. Meta-clustering is proven to be useful to exploratory study cell subsets/states within a cell population in more detail [16, 17, 19]. Meta-clustering analysis revealed 12 clusters with consistently distinct phenotypes (Fig. 1b-d). Overall, 10 of 12 defined clusters (**C1**-**C10**) were positive for both P2Y_12_ and TMEM119, indicating microglial populations (Fig. 1b, c). The other two clusters were a cluster of P2Y_12_^dim^TMEM119^lo/−^CD19^dim^HLA-DR^+^CXCR3^+^CD61^+^ myeloid cells, which was enriched in active lesion compared to NAWM (**C11**, Fig. 1b-e), and one P2Y_12_^−^TMEM119^−^ cluster of mixed CD45^hi^CD66b^+^Clec12A^+^ infiltrating immune cells, which was present at a comparable frequency in NAWM and active lesions (**C12**, Fig. 1c, d). The homeostatic microglial cluster (hoMG, **C3**), which was characterized as P2Y_12_^+^TMEM119^+^CD14^lo^CD68^dim^HLA-DR^dim^CD11c^dim^, was less abundant in active lesions compared to NAWM tissue (Fig. 1c-e). Similarly, we also detected a lower abundance of a unique cluster of P2Y_12_^+^TMEM119^dim^TNF^hi^ microglia (**C8**) in active lesions (Fig. 1c-e). Furthermore, a cluster of P2Y_12_^+^TMEM119^dim^Clec7A^dim^CD14^hi^ activated microglia (**C1**) was detected at higher abundance in active lesions of PMS (Fig. 1c-e). Of note, in each defined cluster, small phenotypic differences were found between myeloid cells in active lesions and NAWM (Supplementary Fig. 6).

**Fig. 2:**
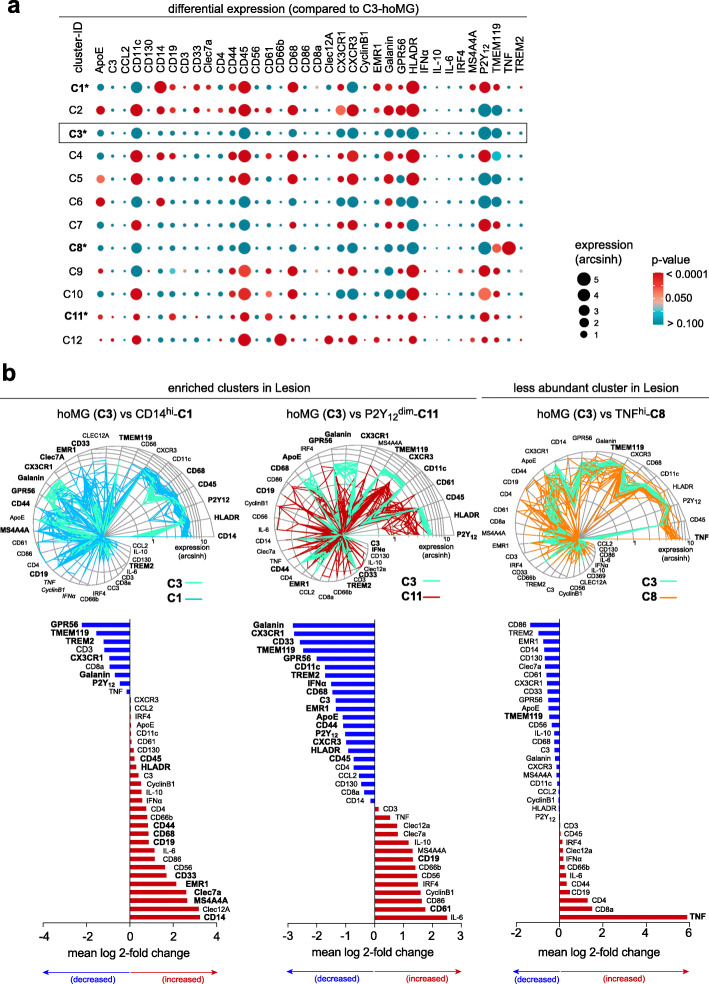
Phenotypic diversity of myeloid cells in active lesions. **a** Heat map of all markers across all profiling clusters, in comparison to hoMG cluster **C3**. Tile size is an expression level (arcsinh) and heat colors show *p*-value (range from < 0.0001 (red) to > 0.100 (green)). **b** Snail plot shows marker expression levels of each differentially abundant clusters (of all samples measured), in comparison to hoMG cluster **C3**. The snail shell represents transverse (perpendicular) axis mapping marker expression levels on an exponential scale. Each line denotes each sample (*n* = 15). The bar plot (below) shows mean (Log 2) fold change of each marker in each differentially abundant cluster, compared to those in hoMG cluster **C3**. Significantly differential expressed markers are in bold. Two-tailed, unpaired t-test followed a correction for multiple comparisons using the Holm-Šídák method. *p*-value < 0.05 is considered statistically significant

Comparing the phenotypes of the three differentially abundant clusters to the homeostatic microglia cluster (hoMG, **C3**) revealed significantly lower expression of microglial markers P2Y_12_, TMEM119, CX3CR1 and GPR56 in both lesion-enriched clusters **C1** and **C11** (Fig. 2a, b). Significantly higher expression of CD45, HLA-DR, CD44, CD68, CD19, CD33, EMR1, Clec7a, MS4A4A and CD14 was detected in the activated microglial cluster **C1** (Fig. 2a, b), whereas only CD19 and CD61 were higher in another P2Y_12_^dim^ myeloid cell cluster **C11** (Fig. 2a, b). Of note, TREM2 expression was found to be lower in these clusters, compared to the hoMG cluster (Fig. 2a, b; Additional file 2: Supplementary Fig. 5). In the less abundant TNF^hi^ microglial cluster **C8**, only TMEM119 and TNF expression was found to be different from the hoMG cluster (Fig. 2a, b). Interestingly, both of the lesion-enriched clusters **C1** and **C11** showed lower expression of microglial homeostatic markers and increased expression of CD19, a B cell marker which has been previously reported in rare cases of human post-mortem microglia sample, compared to the homeostatic cluster [16]. Similar to our previous finding [16], these cells were characterized as CD19^+^CD45^dim^P2Y_12_^dim^HLA-DR^dim^, and thus were phenotypically different from peripheral B cells.

**Fig. 3:**
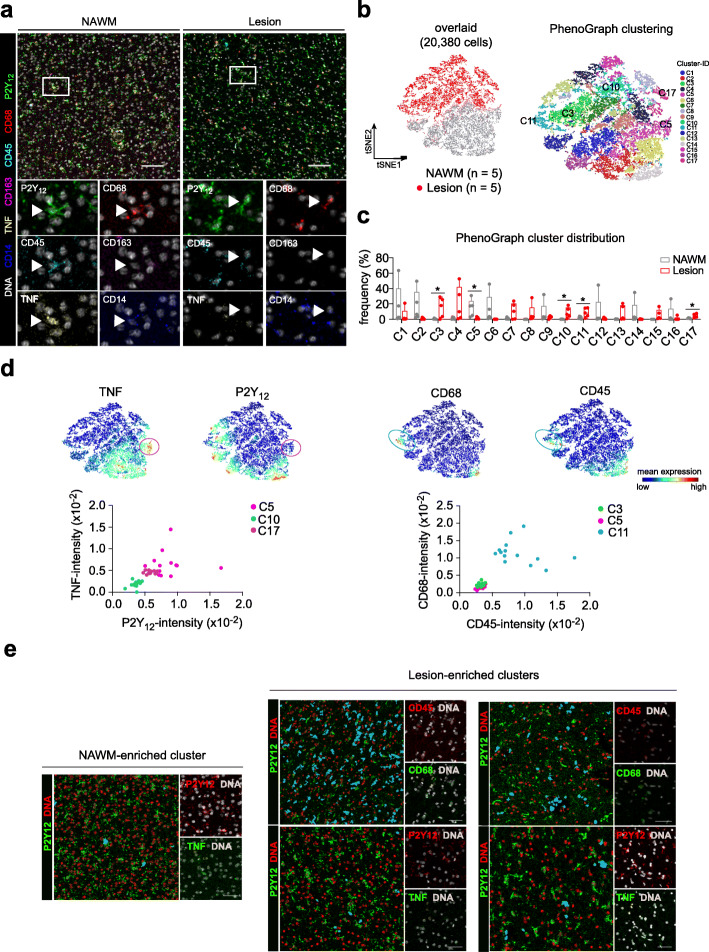
Differentially abundant clusters in active lesions, determined by imaging CyTOF. **a** Representative mass cytometry images of NAWM (*n* = 5 biological replication) and lesion white matter (n = 5 biological replication) (scale bar = 100 μm). Microglia/macrophage are stained with P2Y_12_, CD68, CD45, CD163, CD14 and TNF, and nuclei/DNA are counterstained with Iridium (grey). Arrow head indicates P2Y_12_^+^ microglia, which are positive or negative for TNF. **b** The overlaid t-SNE plot of all segmented cells from all samples. The coloring indicates NAWM (grey dots), Lesion (red dots) or 17 clusters representing diverse myeloid cell phenotypes on the analyzed brain tissues, defined by the PhenoGraph algorithm using an input of k-nearest neighbors of 60. **c** The Box plots shows frequency (%) of all defined clusters. Five differentially abundant clusters (**C3**, **C5**, **C10**, **C11** and **C17**) in lesions, compared to NAWM samples, were labelled with asterisk. Boxes extend from the 25th to 75th percentiles. Whisker plots show the min (smallest) and max (largest) values. The line in the box denotes the median. Each dot represents the value of each sample. Two-tailed, unpaired t-test. *p*-value < 0.05 (*) is considered statistically significant. **d** Reduced-dimensional single-cell t-SNE maps show the expression of TNF, P2Y_12_, CD68 and CD45. Color spectrum indicates expression levels of the marker (red, high expression; blue, low expression). Dot plot shows the correlation between TNF- and P2Y_12_-signal intensity of **C5** (lower abundance in lesions), **C10** and **C17** (lesion-enriched clusters) of all samples measured, and the correlation between CD68- and CD45-signal intensity of **C5** (lower abundance in lesions), **C3** and **C11** (lesion-enriched clusters) of all samples measured. **e** Representative IMC images of five differentially abundant clusters detected in lesion WM (scale bar = 50 μm)

Of note, in contrast to the scRNA-Seq study of small biopsies of MS lesions from patients with early MS [12], this study performed a single-cell protein array of larger brain autopsy tissue containing an active lesion surrounded by NAWM in PMS. To validate whether differentially abundant clusters were indeed located in active lesions, we performed imaging CyTOF (IMC) on formalin-fixed paraffin-embedded (FFPE) tissue blocks from the same donors (Additional file 1: Supplementary Table 1) that were used for CyTOF. IMC simultaneously measures up to 37 proteins at subcellular resolution. This approach allows delineation and quantification of cell heterogeneity in a large area of interest (e.g. 1 mm^2^) [26]. However, due to limitation of commercially available antibodies for IMC and restricted antigen retrieval protocol, we performed the analysis using a panel of 13 antibodies, including those that were analyzed in *Exp-I* (Additional file 1: Supplementary Table 9; Additional file 2: Supplementary Fig. 7a). Tissue microarrays of brain sections (1.5-mm diameter) of all samples (1–3 sections per sample) were generated and stained. A 1-mm^2^ image of each section was taken and analyzed (Additional file 2: Supplementary Fig. 7a). We observed higher abundance of TNF^+^P2Y_12_^+^ microglia in NAWM (Fig. 3a). To further quantify the abundance of this cell subset, P2Y_12_ (a marker defining area of cell cytoplasm) and DNA (^191/193^Ir, a marker defining cell nucleus) signals were used to segment individual cells in each image. The segmented cells (DNA^+^P2Y_12_^+^ and DNA^+^P2Y_12_^−^) were used to perform unsupervised PhenoGraph analysis [27], which partitioned all segmented cells into 17 clusters with distinct phenotypes (Fig. 3b, c; Additional file 2: Supplementary Fig. 7b). Among these clusters, we found five differentially abundant clusters in active lesions, compared to NAWM tissue (Fig. 3c). We could confirm a lower abundance of a cluster of P2Y_12_^+^TNF^+/hi^ cells (similar to **C8** identified by CyTOF (Fig. 2b)) in lesions compared to NAWM (Fig. 3c-e). We also noted a higher abundance of CD45^+/hi^CD68^+/hi^ clusters in active lesions (Fig. 3c-e), which had a similar phenotype to **C1** identified by CyTOF (Fig. 2b). Nevertheless, these lesion-enriched clusters expressed low levels of CD44, CD11c, HLA-DR and CD14 (Additional file 2: Supplementary Fig. 7b), which is similar to the phenotype of **C11** identified by CyTOF (Fig. 2b), indicating that these clusters may contain mixed cells that have similar phenotypes to both **C1** and **C11** identified by CyTOF (Fig. 2b).

### Increased phagocytic phenotypes in lesion-enriched myeloid cells

Microglial activation and phagocytic activity have long been considered key events in MS pathology [28–31]. In *Exp-I*, we found increased expression of markers involved in the clearance of apoptotic cells/bodies including CD61 [32], along with the increased expression of HLA-DR (major histocompatibility (MHC)-II) and phagocytosis-associated markers, such as CD44 and CD68, in the lesion-enriched microglial clusters (Fig. 2a, b), compared to the hoMG cluster. In contrast to results obtained from a mouse model of experimental autoimmune encephalomyelitis (EAE) and from brain biopsies of patients with early MS [12, 13], we did not detect a significant increase in myeloid cell infiltration or strong inflammatory phenotype of microglia in the active lesions of PMS (**C12**, Fig. 1c, d). However, limitations in the antibody panel used in *Exp-I* (Figs. 1 and 2) may have caused the discrepancy with findings from EAE or early MS studies using scRNA-Seq with much higher dimensionality [12, 13]. Furthermore, in *Exp-I* we have also observed a highly phagocytic microglia cluster **C4** (Fig. 2), which was enriched in active lesions but did not reach significant difference compared to NAWM (FDR-adjusted *P* value = 0.0838). This may due to lacking of markers identifying phagocytic and activated states. Therefore, to further investigate the phagocytic and inflammatory phenotypes of myeloid cells, including infiltrating cells, in active lesions of PMS, we used two additional antibody panels (*Exp-II* and *Exp-III;* Additional file 1: Supplementary Table 8). The antibody panels used in *Exp-II* and *Exp-III* had some overlap in phenotypic-defining markers (designated as *TYPE* markers: HLA-DR, CD11c, CCR2, CD172a (SIRPα), CD196, CD91, CD95 (Fas), CD56, CD54 (ICAM-1), CD116, CD74, CD47, IRF7, CD274, CD35). These *TYPE* markers identified different cell subsets/clusters of WM myeloid cells, and allowed us to compare the cell populations between experiments (*Exp-II* and *-III*). In addition, we further phenotypically profiled the defined clusters using *STATE* markers (Additional file 1: Supplementary Table 8), a set of markers characterizing microglia/myeloid cells with particular emphasis on inflammation- and phagocytosis-associated markers, including MIP-1β (CCL4), TNF, GM-CSF, CD206, Clec7a, AXL, CD36, CD163, CD14, CD64 (FcγRI), CD32 (FcγRII), TGF-β, IL-1β, IFNγ, IFNα and osteopontin (OPN; *SPP1*).

**Fig. 4:**
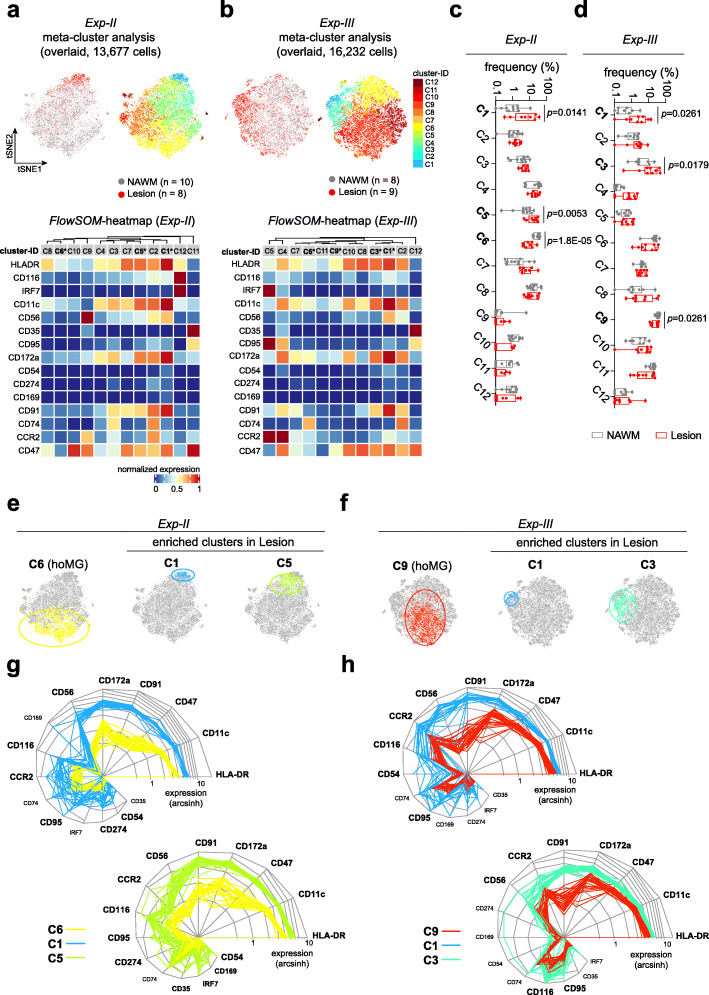
Confirmation of differentially phenotypic diversity of myeloid cells in active lesions. **a, b** The overlaid t-SNE plot of ten NAWM and eight active lesion samples (*Exp-II*) (**a**), or of eight NAWM and nine active lesion samples (*Exp-III*) (**b**). The 2D t-SNE maps were generated based on expression levels of *TYPE* markers of *Exp-II* (Supplementary Table 8**, a**) or of *Exp-III* (Supplementary Table 8**, b**). The coloring indicates 12 defined clusters representing diverse myeloid cell phenotypes. Heat map cluster demonstrates the expression levels of *TYPE* markers used for t-SNE embedding. **c, d** Box-plots show frequencies (%) of all defined clusters in *Exp-II* (**c**) and *Exp-III* (**d**). Differentially abundant clusters (**C1, C5,** and **C6,** *Exp-II;* **C1, C3** and **C9,** *Exp-III*) between active lesions and NAWM were found. A FDR-adjusted *P* value < 0.05 was considered statistically significant, determined using GLMM. Boxes extend from the 25th to 75th percentiles. Whisker plots show the min (smallest) and max (largest) values. The line in the box denotes the median. Each dot represents the value of each sample. **e, f** Reduced-dimensional single-cell t-SNE maps highlight differentially abundant clusters of Exp-II (**e**) and Exp-III (**f**). **g, h** Snail plot shows marker expression levels of each differentially abundant clusters (of all samples), in comparison to hoMG cluster **C6** of *Exp-II* (**g**) or **C9** of *Exp-III* (**h**). Significantly differential expressed markers are in bold. Two-tailed, unpaired t-test followed a correction for multiple comparisons using the Holm-Šídák method. Adjusted *p*-value < 0.05 is considered statistically significant

**Fig. 5:**
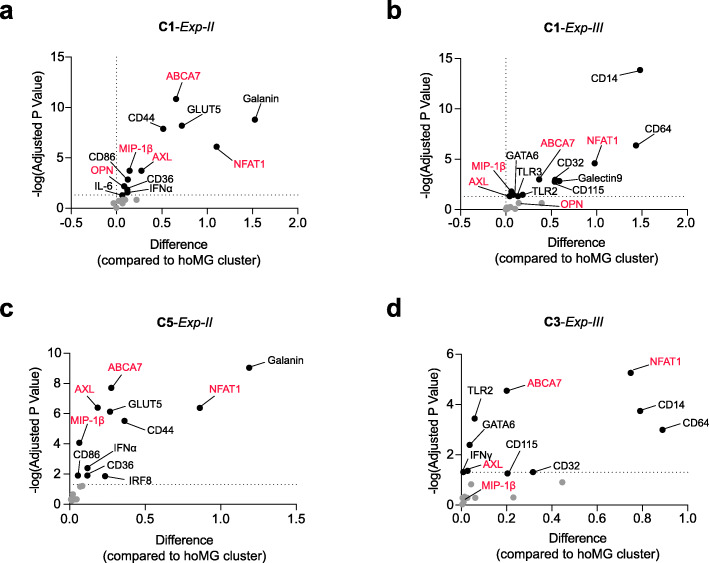
Differential phenotypes of myeloid cells in active lesions. **a-c** Volcano plots show differential expression (in comparison to hoMG) of *STATE* markers (Supplementary Table 8) in significantly enriched microglial clusters, determined using *Exp-II* (**a,c**) and *Exp-III* (**b,d**) antibody panels. Black dots indicate significantly expressed markers, whereas the grey dots are non-significant markers. Markers labelled in red are determined in both *Exp-II* and *Exp-III*. Two-tailed, unpaired t-test followed a correction for multiple comparisons using the Holm-Šídák method. *p*-value < 0.05 is considered statistically significant. The X axis plots the difference in mean expression between the significantly enriched clusters and the hoMG cluster. A dotted grid line is shown at X = 0, no difference. The Y axis plots the multiplicity adjusted p-value (−log) tested using two-tailed, unpaired *t-test*. A dotted grid line is shown at Y = −log (0.05), no statistical significance

As in *Exp-I* (Fig. 1), we first embedded all analyzed cells on t-SNE maps using the *TYPE* markers. Meta-clustering resulted in 12 clusters with consistent phenotypes (Fig. 4a-d; Additional file 2: Supplementary Fig. 8–10). As shown in *Exp-I* (Fig. 1c, d), more than 98% of CD11b-MACS-sorted cells were P2Y_12_^+/dim^TMEM119^+/dim^ microglia. In *Exp-I*, the hoMG cluster was characterized as P2Y_12_^+^TMEM119^+^HLA-DR^dim^CD11c^dim^CD68^dim^. In *Exp-II* and *-III* (in which P2Y_12_ and TMEM119 were not measured, due to limitation of metal channels available), we therefore identified the HLA-DR^dim^CD11c^dim^CCR2^lo/−^ cluster as the hoMG cluster (**C6** in *Exp-II* and **C9** in *Exp-III*). A lower abundance of HLA-DR^dim^CD11c^dim^CCR2^lo/−^ hoMG in active lesions was detected in both *Exp-II* and *-III*, which was similar to results obtained from *Exp-I* (Fig. 4a-f). Increased abundance of two activated microglial clusters was consistently detected in active lesions in both experiments (**C1** and **C5** in *Exp-II*; **C1** and **C3** in *Exp-III*, Fig. 4a-f). These clusters were similarly characterized by higher expression of HLA-DR, CD11c, CD47, CD172a, CD91, CD56, CCR2, CD116 and CD95, compared to the hoMG cluster (Fig. 4g, h). The two activated microglial clusters (**C1** and **C5** in *Exp-II*; **C1** and **C3** in *Exp-III*) displayed similar phenotypes with varying degrees of activation regarding, in particular, the different expression level of HLA-DR, CD11c, CD172a, CD91 and CD47 (Fig. 4g, h). In-depth phenotypic profiling using *STATE* markers revealed significantly increased expression of inflammation- and phagocytosis-associated markers, including NFAT1, MIP-1β (CCL4), CD36, CD44, CD14, CD64 (FcγRI), CD32 (FcγRII), IFNα, AXL, ABCA7, CD115, Toll-like receptors (TLRs), Galanin and GLUT5 in highly activated microglial clusters in active lesions (**C1** in *Exp-II* and **C1** in *Exp-III*, Fig. 5a, b). The clusters with a less activated phenotypes (**C5** in *Exp-II* and **C3** in *Exp-III*, Fig. 5c, b) displayed fewer phenotypic differences in active lesions. We did not detect increased infiltration of CCR2^hi/+^ myeloid cells in active lesions of PMS (Fig. 4a-f; **C2** and **C9** in *Exp-II*; **C4** and **C5** in *Exp-III*), which was in line with the result from *Exp-I* showing no different abundance of Clec12A^+^ myeloid cells in active lesions of PMS (**C12**; Fig. 1c, d).

Similar to results obtained from *Exp-I*, comparing the phenotypes of myeloid cells in active lesions to those in NAWM within the same defined cluster of both *Exp-II* (Supplementary Fig. 11) and *Exp-III* (Supplementary Fig. 12) resulted in small phenotypic differences, with one exception in the case of **C12** (*Exp-II*). In this cluster, strongly reduced expressions of CD116, NFAT1, CD44 and GM-CSF was found in active lesions (Supplementary Fig. 11).

### NAWM microglial phenotypes are comparable to the control aged microglia

**Fig. 6:**
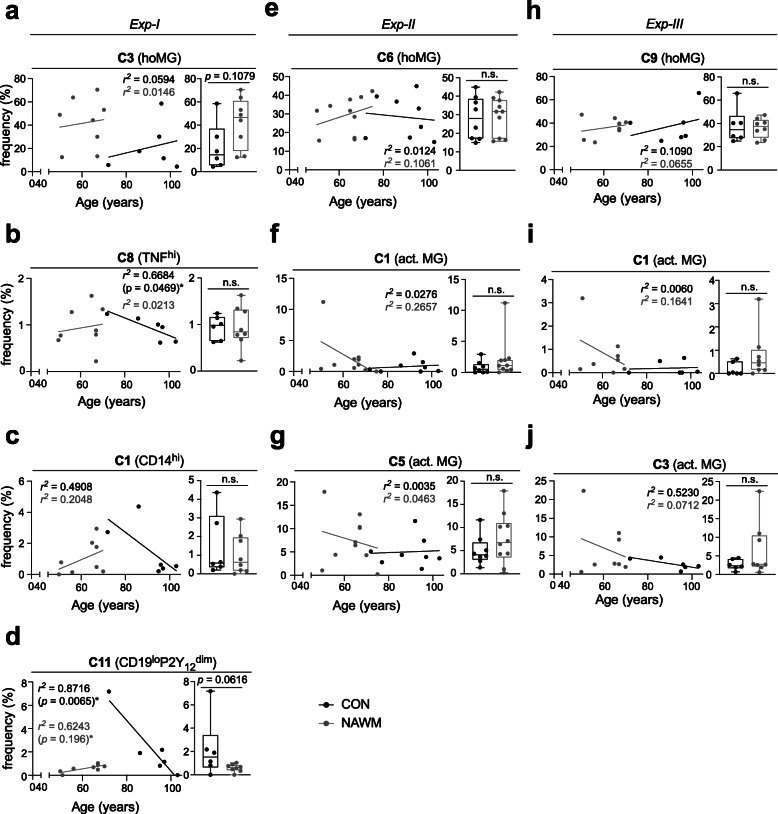
Age-related phenotypic changes of microglia in active lesions. **a-j** Linear regression analysis (*r*^*2*^ denotes the Pearson correlation coefficients) and Box plots compare age and the cluster abundance (frequency, %) of the hoMG clusters and significantly enriched clusters, identified using antibody panels *Exp-I* (**a-d**)*, Exp-II* (**e-g**) and *Exp-III* (**h-j**). Boxes extend from the 25th to 75th percentiles. Whisker plots show the min (smallest) and max (largest) values. A *p* value < 0.05 was considered statistically significant, determined using Mann-Whitney test

Disease onset of primary progressive MS (PPMS) and secondary progressive MS (SPMS) is generally around 10 years later than RRMS [33]. In addition, the incidence of irreversible disability in PPMS and SPMS follows a similar pattern [33], which suggests that aging could be an important risk factor for MS progression. During normal aging, microglia undergo phenotypic and functional changes, resulting in reduced ability to repair CNS damage, which may result in more vulnerable axons and neurons [34]. Furthermore, aged microglia have been observed to display an activated phenotype characterized by increased expression of MHC class II, CD68 and pro-inflammatory cytokines such as IL-1, IL-6 and TNF [16, 35]. This activation is associated with increased expression of TLRs and other pattern recognition receptors, as well as decreased expression of immune-suppressive factors, such as CD200-CD200R and fractalkine-CX3CR1 interactions [35]. Consequently, the active lesion-associated phenotypic changes described above (Figs. 1, 2, 3, 4 and 5) could resemble those observed with aging. In addition, our previous study using bulk transcriptomic analysis revealed subtle changes of the microglial signature in NAWM of PMS donors compared to age-matched non-MS control donors [11], so it is interesting to test whether the phenotypic alterations identified in active lesions (in a comparison to NAWM) can also be detected in non-MS aged WM microglia. Myeloid cells were isolated and MACS-sorted from control white matter as described above, and were characterized using the antibody panels from *Exp-I, −II* and *-III*. In comparison to NAWM, we did not detect differentially abundant clusters in control aged microglia (CON) in either experiment (Fig. 6a-j), which was similar to our results obtained from our previous study comparing microglia isolated from NAWM and age-matched control WM [11]. Furthermore, the lesion-enriched clusters, which were identified in *Exp-I, −II* and *-III* (Fig. 4), were not correlated with age in all three studied groups, except TNF^hi^ **C8** (*Exp-I*, Fig. 6b) and CD19^lo^P2Y_12_^dim^ **C11** clusters (*Exp-I*, Fig. 6d).

## Discussion

In this study, we characterized and compared WM myeloid cells isolated from active lesions and NAWM of ten PMS donors, using single-cell mass cytometry to analyse three different antibody panels (a total of 74 markers). Depending on a set of markers analyzed, we consistently detected a lower abundance of a cluster of P2Y_12_^+^TMEM119^+^ and/or HLA-DR^dim^CD11c^dim^ hoMG in active lesions and significantly enriched clusters of highly phagocytic and activated microglia. These clusters were mainly characterized by lower expression of homeostatic markers CX3CR1, P2Y_12_, TMEM119, GPR56, and/or increased expression of proteins involved in phagocytic activity and microglial activation including CD45, HLA-DR, CD44, CD14, CD11c, CD68, Clec7a, MS4A4A, CCR2, CD64, CD32, CD47, CD91, CD95, NFAT1, AXL, ABCA7 and/or cytokine MIP-1β (CCL4) and osteopontin (OPN, or *SPP1*). Our findings are greatly complementary to the results of previous transcriptomic studies in EAE and early MS at the level of single-cell proteomics [12, 13]. Importantly, the abundance of infiltrating myeloid cells was not increased in active lesions of PMS in all three experiments.

In contrast to scRNA-Seq, a single-cell protein array using CyTOF is often limited to a maximum of 40 markers per measurement, and thus it is challenging to comprehensively characterize a targeted cell population with only one antibody panel. We applied three different antibody panels to increase the capacity for in-depth phenotypic profiling. We demonstrated the use of *TYPE* markers to compare differentially abundant clusters between measurements. Moreover, this study underscored the feasibility of performing single-cell phenotypic screening of small microglia/macrophage samples (3 measurements of 10^5^ cells), as we have demonstrated previously [16]. To identify differentially abundant clusters between conditions, we applied meta-clustering analysis (the FlowSOM algorithm: *FlowSOM/Consensus-ClusterPlus*) [19–21], a powerful tool to explore cellular heterogeneity. However, the identified clusters are descriptive and could be interpreted as distinct cell subsets and/or transient cell states. Further functional analysis of each identified cluster remains to-date exceedingly challenging but essential. In addition to CyTOF, we performed imaging CyTOF (IMC) to validate the results obtained from single-cell suspension samples. To date, IMC has been applied to identify multiple myeloid phenotypes on highly inflamed active lesions from one [36] or two [37] patients with RRMS. In this study, using IMC we could confirm phenotypic changes of myeloid cells characterized by CyTOF in active lesions compared with NAWM (from a total of seven PMS donors, Fig. 3; Additional file 1: Supplementary Table 1). However, a direct comparison between the two CyTOF technologies remains technically challenging, due to for example a big difference in the area of analysis (tissue weight of ~ 1 g (CyTOF) vs 1-mm^2^ image (IMC)), differences in cell types analyzed, tissue/cell quality (MACS-pre-isolated single-cell from enzymatically digested fresh post-mortem tissue (CyTOF) vs formalin-fixed, paraffin-embedded tissues (IMC)) or the number of antibodies compatible in one staining protocol that includes antigen-retrieval process, which would limit the dimensionality of the IMC data.

Myeloid cells including microglia are emerging as key players in neuroinflammatory diseases like MS [38–40]. Numerous findings in rodent models such as EAE highlight the importance of myeloid cells including microglia, monocyte-derived macrophages and dendritic cells in neuroinflammation [39, 41]. However, these models only partially replicate the complexity of human MS and thus, our understanding of how myeloid cells either respond or contribute to MS pathogenesis is still limited. This is particularly true for the advanced stages of MS where progressive neurodegeneration predominates [42]. The present study nicely complements the single-nuclei RNA-sequencing study from WM in SPMS [43] and other studies using bulk and single-cell/nuclei transcriptomic analysis in early and PMS as well as the EAE model [12, 13, 41, 44–47], in which microglia show an increased gene expression of MHC class II-related molecules such as *HLA-DR*, *Cd74* and molecules involved in phagocytosis and/or myelin uptake including *GPNMB*, *SPP1* and *Cd68* in MS. Using CyTOF, we detected decreased abundance of the homeostatic microglial cluster in lesion-enriched microglia populations, which consistently coincided with increased expression of the antigen-processing and phagocytosis-related markers HLA-DR, CD11c, AXL, CD45, CD68, ATP-binding cassette (ABC) transporter A7 (ABCA7) [48] and CD44 (a receptor of GPNMB) [49], as well as the molecules involved in the inflammatory process in microglia such as CD14 and Clec7a (dectin-1) and its co-activator MS4A4A [50]. Expanding the analyzed markers with *Exp-II* and *-III* revealed active lesion-enriched clusters of cells with higher expression of phagocytosis-related and inflammatory molecules such as inflammatory cytokines MIP-1β (CCL4) and OPN, the receptor tyrosine kinase AXL, the myeloid inhibitory immunoreceptor SIRPα (CD172a) and its co-activator CD47 [51], ABCA7, CD91 (LRP1 or ApoE receptor) and Fcγ receptors (CD64 and CD32). Furthermore, the expression of molecules involved in apoptosis-regulation CD95 (Fas) and immune regulatory function NFAT1 (a transcription factor regulating T-cell function) and galanin [52, 53] were also found to be increased in lesion-enriched clusters. Even though our study lacks functional investigation, it is tempting to speculate that, at this late disease stage of PMS, microglia are multi-functional. On the one hand, microglia attempt to maintain brain homeostasis by up-regulating expression of molecules involved in clearance of apoptotic cells and myelin debris such as AXL [54], phospholipid transporter ABCA7 [48], HLA-DR, CD45 and CD68, as well as the neuropeptide galanin, which provides neuroprotective effect in EAE mouse model [53]. On the other hand, some microglia become activated and up-regulate the expression of inflammatory mediators MIP-1β and OPN. An expansion of MIP-1β (*Ccl4*)-expressing microglia subset has been detected in EAE [13] and MS active lesions [12], and was proposed to be a neurotoxic population. Similarly, OPN (*SPP1*) is involved in microglia activation pathway and has been found up-regulated in EAE [13] and WM active lesions of MS patients [12], as well as in an Alzheimer’s disease model [55]. It has also been demonstrated in a mouse model of demyelination that OPN exacerbated disease progression, promoted worsening paralysis and induced neurological deficits [56]. Furthermore, the increased expression of the immune regulator NFAT1 in microglia in active lesions may be linked to chronic activation and neuroinflammation of these cells [52, 57].

It has been challenging to distinguish microglia from infiltrating macrophages in human brain and thus recognize their contribution to MS lesion formation and pathology. In this study, to distinguish microglia from infiltrating macrophages in active MS lesions, we used either microglia signature markers P2Y_12_, TMEM119 and GRP56, together with Clec12A, a marker for hematogenic macrophage (*Exp-I*) or CD14 and CCR2 for monocytes and/or monocyte-derived macrophages (*Exp-II* and *-III*) [58]. Interestingly, the cluster with high Clec12A expression and low P2Y_12_, TMEM119 and GPR56 expression was not significantly enriched in active MS lesions. Similarly, the abundance of CCR2^hi/+^ cells was comparable between NAWM and active lesions. Our findings are similar to a previous study performed in a mouse model of demyelination, in which microglia became activated in response to lysophosphatidylcholine (LPC)-induced demyelination, and dominated the CNS lesion by limiting the dispersion of CNS-infiltrating macrophages into the lesioned WM [59]. On the contrary, recent studies using scRNA-Seq have demonstrated that monocyte-derived macrophages can enter the CNS during early MS or EAE [12, 41]. This discrepancy in findings may be due to differences in studied models/diseases (e.g. early MS vs PMS; EAE vs LPC-induced demyelination) or an analytical method used (scRNA-Seq vs CyTOF), which may lead to differences in cell identification/clustering. A direct comparison between studied models/diseases using a single analytical method is required to make a meaningful conclusion.

We detected a distinct cluster of P2Y_12_^+^ microglia that highly expressed TNF (Fig. 1e). Interestingly, a lower abundance of this TNF^hi^ microglial cluster was found in active lesions of PMS, compared to NAWM. TNF has long been recognized as an immune modulator [60]. During neuroinflammation, TNF is mainly expressed by myeloid cells [61, 62], and provides neuroprotective effects, possibly by limiting the extent and severity of autoimmune pathology [60–63]. TNF deficiency is related to disturbed microglial homeostasis [61], suggesting an important role of TNF in microglia function. This concept is supported by the results obtained from the EAE model, indicating that impairment of TNF signaling is associated with the induction of demyelination and less removal of T lymphocytes from the lesion area [64]. Moreover, monoclonal antibody therapies targeting TNF and its receptors (TNFRs) have been shown to potentially induce demyelinating disorders in human [65]. It has been also demonstrated that lipid uptake in microglia induced non-inflammatory phenotype by downregulating TNF expression [66]. Together, TNF-TNFRs signaling may play an important role in maintaining homeostatic function of microglia. However, it is technically impossible to selectively sort this rare population, thus their precise function in PMS remains to be investigated.

## Conclusions

In summary, we demonstrate herein the power of multi-dimensional single-cell phenotyping to unravel the diversity of myeloid cells in PMS post-mortem brain tissue. Our results underscore the heterogeneity and complexity of myeloid cell phenotypes in active lesions of PMS, and suggest potential differences of pathogenesis between early MS and PMS. Active lesions of PMS contain highly phagocytic and activated microglia, pointing towards their role in clearing up myelin/cellular debris without being fully activated by the lesion environment. This may explain why anti-inflammatory therapies that are highly effective in early MS are less effective in PMS. It will be important to consider the heterogeneity of myeloid cell phenotypes when designing novel treatment interventions for PMS.

## Supplementary information

**Additional file 1.** Supplementary tables referred to in the article text.

**Additional file 2.** Supplementary figures referred to in the article text.

## Data Availability

The datasets used and/or analyzed during the current study are available from the corresponding author on reasonable request that does not include confidential patient information.
