# Supplementary material for: Single-cell mass cytometry reveals complex myeloid cell composition in active lesions of progressive multiple sclerosis
Source: Acta Neuropathol Commun. 2020 Aug 18;8:136. doi: 10.1186/s40478-020-01010-8 (PMC7437178; doi:10.1186/s40478-020-01010-8)
Supplement: Supplementary file 1 — Additional file 1. Supplementary tables referred to in the article text. [file 40478_2020_1010_MOESM1_ESM.pdf]

Supplementary Table 1 Donor characteristics

Diagn.	Gender	Age	PMD	pH CSF	Time until processing	Cause of death	Disease duration	Time to EDSS6	Tissue	Exp./ Analysis
CON	M	92	8:50	6.89	19:20	Pneumonia	-	-	-	II**
CON	F	77	8:30	6.21	18:30	Died during femur fracture operation	-	-	-	II**
CON	F	103	9:50	NA	22:10	Heart failure	-	-	-	I, II, III
CON	M	97	5:10	6.65	12:40	Kidney failure	-	-	-	I, II, III
CON	M	86	6:45	6.39	14:10	Complications due to leaking EVAR prosthesis	-	-	-	I, II, III
CON	F	95	4:21	6.59	9:10	Ileus caused by tumor	-	-	-	I, II, III
CON	F	96	8:30	6.70	NA	Stopped drinking & eating	-	-	-	I, II, III
CON	M	72	6:55	6.81	NA	Multiple myeloma and renal insufficiency	-	-	-	I, II, III
PPMS	M	75	9:05	NA	5:10	Euthanasia	42	31	NAWM	II**, IMC
SPMS	F	65	10:20	6.56	9:20	Pneumonia	37	14	NAWM	II**
SPMS	M	65	9:30	6.50	8:00	Euthanasia	34	8	NAWM & Lesion	I, II, III, IMC
SPMS	F	51	9:10	6.87	4:45	Euthanasia	17	16	NAWM & 2x Lesion	I, II, III, IMC, Flow
SPMS	M	70	9:25	7.22	9:50	Euthanasia	33	20	NAWM & 2x Lesion	I, II, III, IMC, Flow
SPMS	F	67	9:20	NA	13:30	Pneumonia	25	11	NAWM & Lesion	I, II, III, IMC, Flow
PPMS*	F	67	5:45	6.62	13:10	Euthanasia	6	8	NAWM & Lesion	I, II, III, IMC, Flow
PPMS*	M	67	7:55	6.40	23:50	Euthanasia	11	5	NAWM & Lesion	I, II, III, IMC, Flow
SPMS*	F	50	10:15	6.44	7:35	Euthanasia	13	11	NAWM & 2x Lesion	I, II, III, Flow
SPMS*	M	56	6:15	6.60	20:15	Pneumonia	19	7	NAWM	I, II, III, Flow

Diagn. = diagnosis; PMD = post-mortem delay (hr:min); CSF = cerebrospinal fluid; CON = non-MS control donors; SPMS = secondary progressive multiple sclerosis; PPMS = primary progressive multiple sclerosis; F = female; M = male; NA = not available; NAWM = normal-appearing white matter; IMC = imaging CyTOF; Flow = flow cytometry; Time until processing= time tissue was stored in Hibernate-A medium until processing (hrs:min); *MS diagnosis based on clinical data, diagnosis is not yet confirmed by neuropathologist; **Due to low cell number, these donors can be analyzed only in one experiment (*Exp-II*)

Supplementary Table 2 Summary of donor characteristics

Diagn.	*N*	F/M	Age (yr)	PMD (hr:min)	pH CSF	Disease Duration (yr)	Time to EDSS 6 (yr)
CON	8	4/4	89.75 ± 10.63	7:40 ± 1:42	6.69 ± 0.19	-	-
MS	10	5/5	63.30 ± 8.23	6:42 ± 1:34	6.65 ± 0.27	24.7 ± 11	13.1 ± 7.8
p value (unpaired *t-test*)		>0.9999	<0.0001	0.2369	0.7973	-	-

Diagn. = diagnosis; PMD = post-mortem delay (hr:min); CSF = cerebrospinal fluid; CON = control donors; MS = multiple sclerosis donors; F = female; M = male

Supplementary Table 3 **Donor characteristics – IRF8+ nuclei analysis by qPCR**

NBB no.	Tissue	Diagn.	Gender	Age	PMD	pH CSF	Cause of death	Disease duration
1996-026	NAWM	SPMS	F	69	09:15	6.40	General decline due to MS	19
1998-158	NAWM	SPMS	F	76	14:15	5.93	Respiratory insufficiency	34
1999-073	NAWM	MS	F	71	08:20	6.80	Pneumonia	24
1999-119	NAWM	RRMS	F	38	05:15	6.55	Sudden death, unknown cause	9
2000-024	NAWM	PPMS	F	52	08:25	6.10	Respiratory failure during pneumonia	12
2001-093	NAWM	SPMS	F	66	06:20	6.44	Cancer metastasis in liver	43
2012-056	NAWM	SPMS	M	78	08:45	6.41	Euthanasia	33
1999-109	Active lesion	SPMS	M	70	06:25	6.28	Pneumonia	22
2001-135	Active lesion	SPMS	M	43	08:30	6.48	Pneumonia	26
2007-085	Active lesion	SPMS	F	66	06:00	6.18	unknown	23
2010-025	Active lesion	SPMS	M	51	11:00	6.23	unknown	16
2001-018	Active lesion	SPMS	F	48	8:10	6:55	Euthanasia	8
p-value (unpaired *t-test*)			0.2222	0.2936	0.6815	0.8289	-	0.5536

Age = age at death (years); CSF = cerebrospinal fluid; Disease duration (years); F = female; M = male; PPMS = primary progressive MS, RRMS = relapsing-remitting MS; SPMS = secondary progressive MS; NAWM = normal-appearing white matter; NBB no. = donor registration number of the Netherlands Brain Bank; PMD = post-mortem delay (h:min).

Supplementary Table 4 Primer pairs used for RT-qPCR analysis of IRF8$^+$ nuclei

Gene symbol	Forward sequence (5´-3´)	Reverse sequence (5´-3´)
ADGRG1	CTCTCCTAAGAGGTTCTCTCCA	CTACAACAGGCCAGCAATCTA
CX3CR1	GGACTGTGTTCCTGTCCATATT	GTGACACTCTTGGGCTTCTT
EEF1A	AAGCTGGAAGATGGCCCTAAA	AAGCGACCCAAAGGTGGAT
GAPDH	TGCACCACCAACTGCTTAGC	GGCATGGACTGTGGTCATGA
P2RY12	CTGTGCACCAGAGACTACAA	GCCAGGCCATTTGTGATAAG
TMEM119	CAACTTCCTGGATGGGATAGTG	AGACGATGAACATCAGCAGAAA

Supplementary Table 5 List of antibodies used for flow cytometry

target	clone / company	fluorochrome	dilution
CD3	SK7 / BD Biosciences	PerCP-Cy5.5	1:25
CD11b	ICRF44 / eBioscience	PE<br>PE-Cy7	1:200
CD19	SJ25C1 / BD Bioscience	PerCP-Cy5.5	1:20
CD45	HI30 / BD Biosciences	BB515	1:200
CD56	HCD56 / BioLegend	PerCP-Cy5.5	1:100
CD66b	G10F5 / BioLegend	PerCP-Cy5.5	1:100
CX3CR1	2A9-1 / eBioscience	PE-Cy7	1:200
GPR56	CG4 / BioLegend	PE-Cy7	1:100
$P2Y_{12}$	S16001E / BioLegend	PE-Cy7	1:100
fixable viability dye eFluor™ 506	-	eFluor™ 506	1:500

Supplementary Table 6 Donor characteristics

NBB no.	Diagn.	Gender	Age	PMD	pH CSF	Cause of death	Disease duration	Tissue
10-015	CON	F	73	07:45	NA	Palliative sedation in end stage metastasized mamma carcinoma	-	WM
11-091	CON	M	76	06:45	6.31	Lung cancer	-	WM
12-002	CON	M	55	07:15	NA	Intestinal ischemia	-	WM
15-055	CON	F	72	06:50	7.22	Euthanasia and metastatic ovarian cancer	-	WM
17-005	CON	F	60	05:30	7.07	Euthanasia	-	WM
01-137	SPMS	M	81	8:50	6.33	Marginal intake of food, drinks and medication	51	Active lesion
06-045	SPMS	M	56	8:00	6.65	Pneumonia	21	Active lesion
10-034	SPMS	F	57	8:40	6.05	Respiratory insufficiency by (uro)sepsis	27	Active lesion
12-084	SPMS	M	63	8:15	8:15	Pneumonia, cachexia and dehydration	27	Active lesion
p-value (unpaired *t-test*)		0.5238	0.6812	0.0093	0.9098	-	-	-

Age in years, Diagn. = diagnosis; Disease duration in years, PMD = post-mortem delay (hr:min); CSF = cerebrospinal fluid; CON = control donors; MS = multiple sclerosis donors; SPMS = secondary progressive multiple sclerosis; F = female; M = male; NA = not available; NBB= Netherlands brain bank; WM= white matter

Supplementary Table 7 List of all CyTOF antibodies and reagents used

target	clone / company	catalogue no.	dilution
ABCA7	polyclonal / Merck	ABC349	1:50
ApoE	WUE-4 / Novus Biologicals	NB110-60531	1:100
AXL	polyclonal / Lifespan Biosciences	LS-C100246-400	1:50
CCL2	5D3-F7 / Biolegend	502601	1:100
CCR2	K036C2 / Biolegend	357202	1:100
CD3	UCHT1 / Fluidigm	3154003B	1:100
CD4	RPA-T4 / Biolegend	300541	1:100
CD8a	RPA-T8 / Fluidigm	3162015B	1:100
CD11c	Bu15 / Fluidigm	3147008B	1:100
CD14	RMO52 / Fluidigm	3160006B or 3148010B	1:100
CD16	3G8 / Biolegend	302002	1:100
CD19	HIB19 / Fluidigm	3142001B	1:100
CD32	6C4 / eBioscience	11-0329-42	1:100
CD33	WM53 / Fluidigm	3169010B	1:100
CD35	594708 / R&D Systems	MAB5748	1:100
CD36	5-271 / Biolegend	336215	1:100
CD38	HIT2 / Fluidigm	3144014B	1:100
CD44	BJ18 / Biolegend	338802	1:20
CD45	HI30 / Fluidigm	3141009B	1:100
CD47	CC2C6 / Fluidigm	3209004B	1:100
CD54 (ICAM1)	HA58 / Biolegend	353102	1:100
CD56	B159 / Fluidigm	3155008B	1:100
CD56	NCAM16.2 / Fluidigm	3149021B	1:100
CD61	VI-PL2 / Fluidigm	3209001B	1:100
CD64	10.1 / Fluidigm	3146006B	1:100
CD66b	80H3 / Fluidigm	3152011B	1:100
CD68	Y1/82A / Biolegend	333802	1:100
CD74	LN2 / Fluidigm	3166018B	1:50
CD86	IT2.2 / Biolegend	305435	1:100
CD91	A2MR-α2 / BD Bioscience	550495	1:100
CD95	DX2 / Biolegend	305602	1:100
CD115	9-4D2-1E4 / Biolegend	347302	1:100
CD116	4HI / Biolegend	305902	1:100
CD130	2E1B02 / Fluidigm	3168016B	1:100
CD141	1A4 / Fluidigm	3173002B	1:100
CD163	GHI/61 / Biolegend	333602	1:100
CD169	CD169 / Fluidigm	3158027B	1:100
CD172a	15-414 / Biolegend	372102	1:100
CD206	15-2 / Fluidigm	3168008B	1:100
CD274	29E.2A3 / Fluidigm	3156026B	1:100
CD369	15E2 / Biolegend	355402	1:50
Clec12A	50C1 / Fluidigm	3173007B	1:100
Complement C3c	4C6A8 / Novus Biologicals	NBP2-61855	1:50
CyclinB1	GNS-1 / Fluidigm	3153009A	1:100

Supplementary Table 7 List of all CyTOF antibodies and reagents used - *continued*

target	clone / company	catalogue no.	dilution
CXCR3	G025H7 / Fluidigm	3163004B	1:100
CX3CR1	2A9-1 / Fluidigm	3172017B	1:50
EMR1	A10 / Bio-Rad	MCA2674GA	1:100
Galanin	581403 / Fluidigm	3148016B	1:100
Galectin-9	9M1-3 / Fluidigm	3163002B	1:100
GATA6	D61E4 / Fluidigm	3161005A	1:100
Glut5	195205 / R&D Systems	MAB1349	1:100
GM-CSF	BVD2-21C11 / Biolegend	502315	1:100
GPR56	CG4 / Biolegend	358202	1:50
HLA-DR	L243 / Fluidigm	3143013B	1:100
IFNα	LT27:295 / Miltenyi Biotec	130-099-214	1:10
IFNγ	B27 / Fluidigm	3168005B	1:100
IL-1β	H1b-98 / Biolegend	511703	1:100
IL-6	MQ2-13A5 / Biolegend	501101	1:100
IL-10	JES3-9D7 / Fluidigm	3166008B	1:20
IRF4	IRF4.3E4 / Biolegend	646402	1:100
IRF7	12G9A36 / Biolegend	656002	1:50
IRF8	7G11A45 / Biolegend	656502	1:100
MIP-1β	D21-1351 / Fluidigm	3150004B	1:100
MS4A4A	818112 / R&D Systems	MAB7797	1:50
NFAT1	D43B1 / Fluidigm	3143023A	1:100
OPN	polyclonal / Lifespan Biosciences	LS-C99283-400	1:100
$P2Y_{12}$	polyclonal / Sigma-Aldrich	HPA014518	1:100
TGF-β	TW4-2F8 / Biolegend	349602	1:100
TIM3	F38-2E2 / Biolegend	345019	1:100
TLR2	TL2.1 / Fluidigm	3176021B	1:100
TLR3	TLR3.7 / eBioscience	13-9039-80	1:50
TMEM119	A16075D / Biolegend	853302	1:10
TNF	Mab11 / Fluidigm	3146010B	1:100
TREM2	237920 / R&D Systems	MAB17291	1:50
DNA	- / Fluidigm	201192A	1:1000
DNA	- / Fluidigm	201192A	1:1000

Supplementary Table 8 List of antibodies for *Exp-I, -II and III*

isotope tag	target		
	Exp-I	***Exp-II***	***Exp-III***
^{141}Pr	CD45	HLA-DR (T)	HLA-DR (T)
^{142}Nd	CD19	CD116 (T)	CD116 (T)
^{143}Nd	HLA-DR	NFAT1	NFAT1
^{144}Nd	CD44	CD44	CD38
^{145}Nd	CD4	IRF7 (T)	IRF7 (T)
^{146}Nd	TNF	TNF	CD64
^{147}Sm	CD11c	CD11c (T)	CD11c (T)
^{148}Nd	Galanin	Galanin	CD14*
^{149}Sm	CCl2	CD56 (T)	CD56 (T)
^{150}Nd	CD86*	MIP-1β	MIP-1β
^{151}Eu	CD68	CD35 (T)	CD35 (T)
^{152}Sm	CD66b	CD95 (T)	CD95 (T)
^{153}Eu	CyclinB1	IL-6*	TIM3
^{154}Sm	CD3	CD172a (T)	CD172a (T)
^{155}Gd	CD56	CD54 (T)	CD54 (T)
^{156}Gd	IL-6*	CD274 (T)	CD274 (T)
^{158}Gd	CD369	CD169 (T)	CD169 (T)
^{159}Tb	IRF4	GM-CSF	GM-CSF
^{160}Gd	CD14*	CD163	CD32
^{161}Dy	EMR1	CD36	GATA6
^{162}Dy	CD8a	CD91 (T)	CD91 (T)
^{163}Dy	CXCR3	Glut5	Galectin9
^{164}Dy	TMEM119	-	CD115
^{165}Ho	$P2Y_{12}$	IRF8	CD16
^{166}Er	IL-10	CD74 (T)	CD74 (T)
^{167}Er	MS4A4A	ABCA7	ABCA7
^{168}Er	CD130	CD206	IFNγ
^{169}Tm	CD33	TGF-β	TGF-β
^{170}Er	GPR56	CD86*	TLR3
^{171}Yb	TREM2	CCR2 (T)	CCR2 (T)
^{172}Yb	CX3CR1	OPN	OPN
^{173}Yb	Clec12A	Clec12A	CD141
^{174}Yb	Complement C3c	IL-1β	IL-1β
^{175}Lu	ApoE	AXL	AXL
^{176}Yb	IFNα	IFNα	TLR2
^{209}Bi	CD61	CD47 (T)	CD47 (T)

T = Type marker; * denotes the markers that are labelled with different metal-isotopes.

Supplementary Table 9 List of imaging CyTOF antibodies

isotope tag	target	clone / company	catalogue No.	dilution
^{144}Nd	CD14	EPR3653 / Fluidigm	3144025D	1:50
^{146}Nd	CD16	EPR16784 / Fluidigm	3146020D	1:50
^{147}Sm	CD163	EDHu-1 / Fluidigm	3147021D	1:50
^{149}Sm	CCR4	L291H4 / Fluidigm	3149030D	1:50
^{150}Nd	CD44	156-3C11 / Bio-Rad	MCA2726	1:50
^{152}Sm	CD45	D9M8I / Fluidigm	3152018D	1:100
^{154}Sm	CD11c	polyclonal / Fluidigm	3154025D	1:50
^{158}Gd	Clec7a	Polyclonal / Lifespan Biosciences	LS-B8826-0.05	1:50
^{159}Tb	CD68	KP1 / Fluidigm	3159035D	1:250
^{165}Ho	$P2Y_{12}$ (biotin-conjugated)<br>biotin	polyclonal/ Sigma-Aldrich<br>1D4-C5 / Fluidigm	HPA014518<br>3165012B	1:100<br>1:100
^{166}Er	CD74	LN2 / Fluidigm	3166025D	1:50
^{172}Yb	TNF	M1-C4 / Sigma-Aldrich	SAB1404480-100UG	1:50
^{174}Yb	HLA-DR	YE2/36 HLK / Fluidigm	3174023D	1:50
^{191}Ir	DNA	- / Fluidigm	201192A	1:1000
^{193}Ir	DNA	- / Fluidigm	201192A	1:1000
