# Supplementary material for: Single-cell mass cytometry reveals complex myeloid cell composition in active lesions of progressive multiple sclerosis
Source: Acta Neuropathol Commun. 2020 Aug 18;8:136. doi: 10.1186/s40478-020-01010-8 (PMC7437178; doi:10.1186/s40478-020-01010-8)
Supplement: Supplementary file 2 — Additional file 2. Supplementary figures referred to in the article text. [file 40478_2020_1010_MOESM2_ESM.pdf]

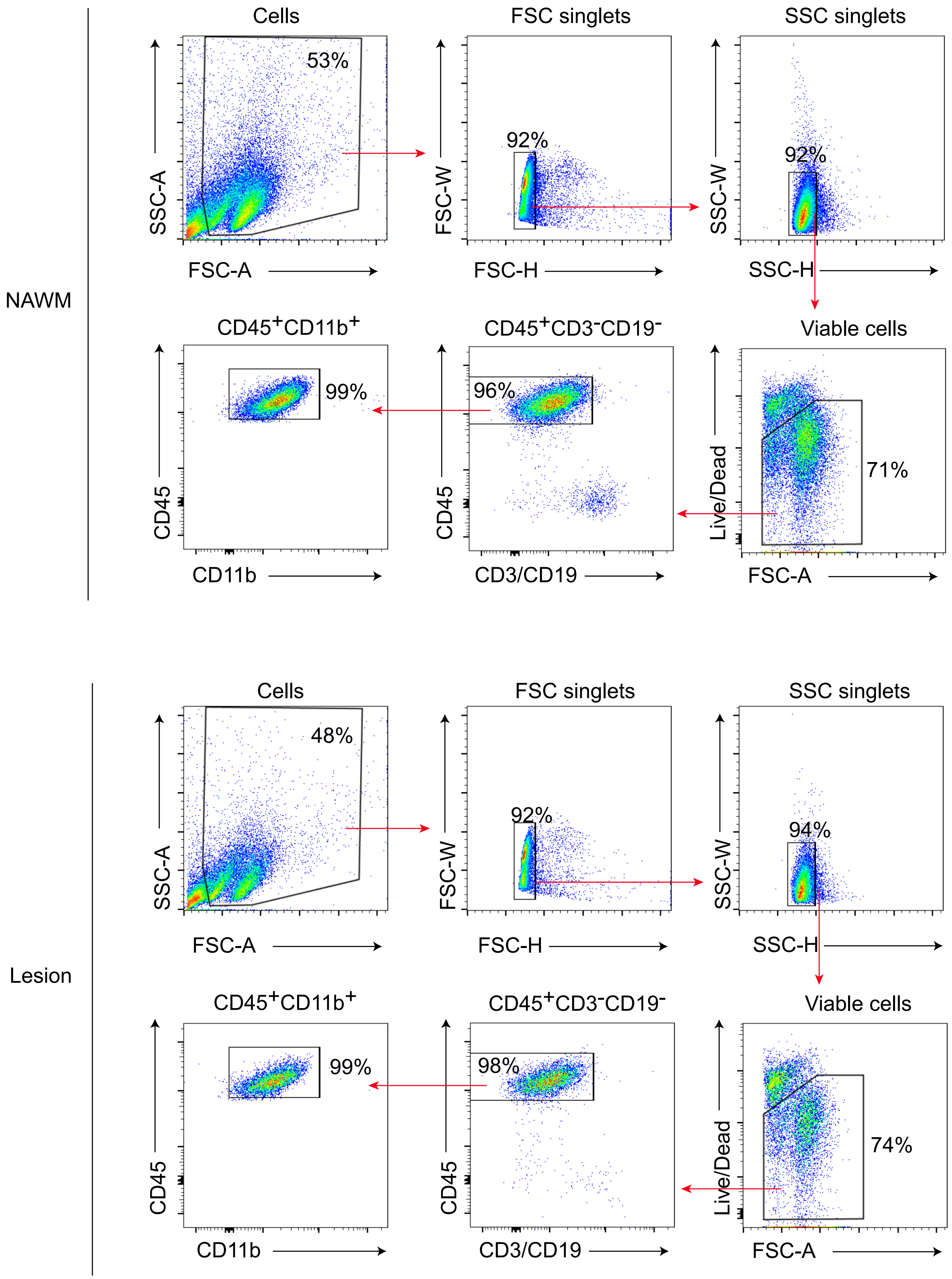

Supplementary Figure 1

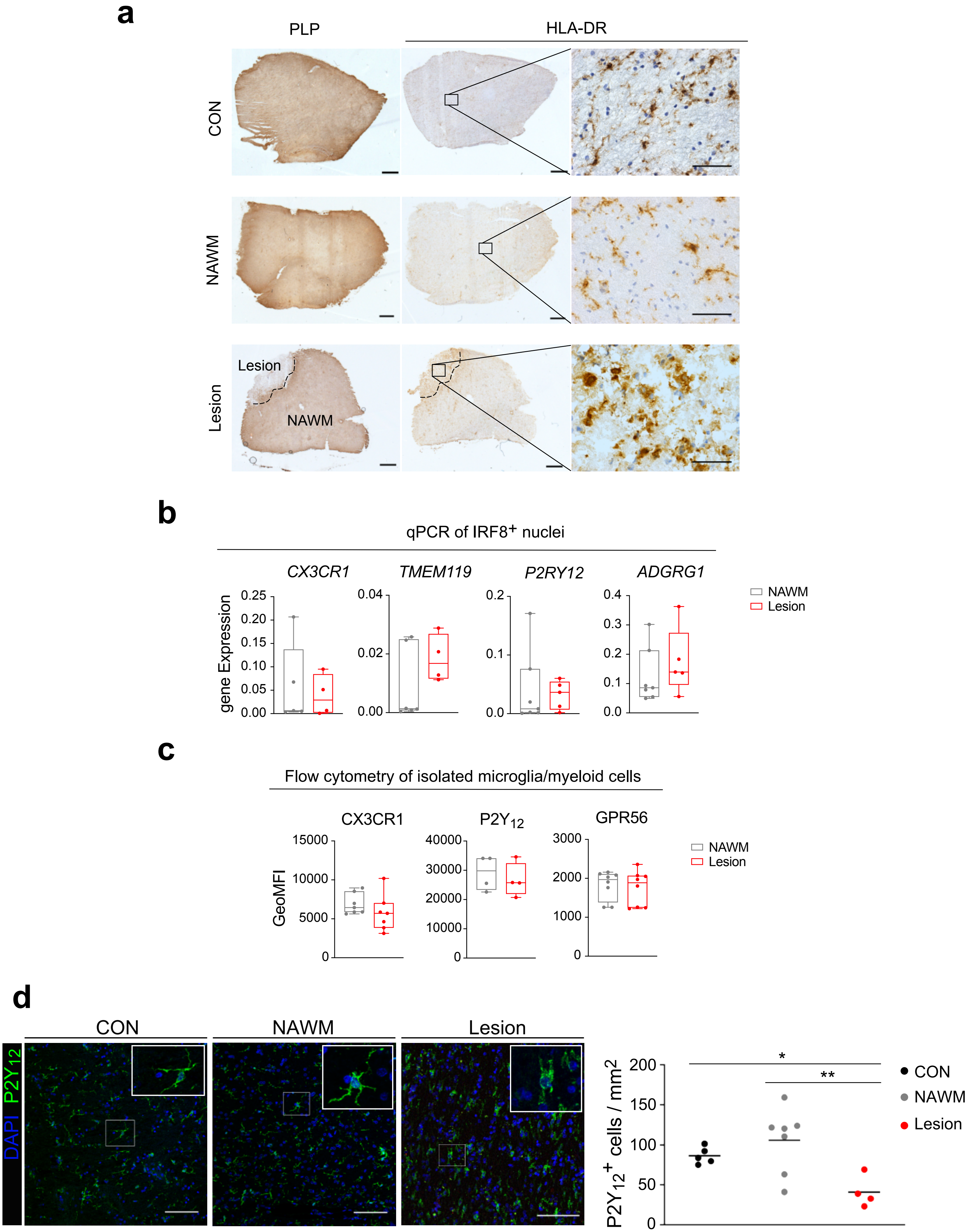

Supplementary Figure 2

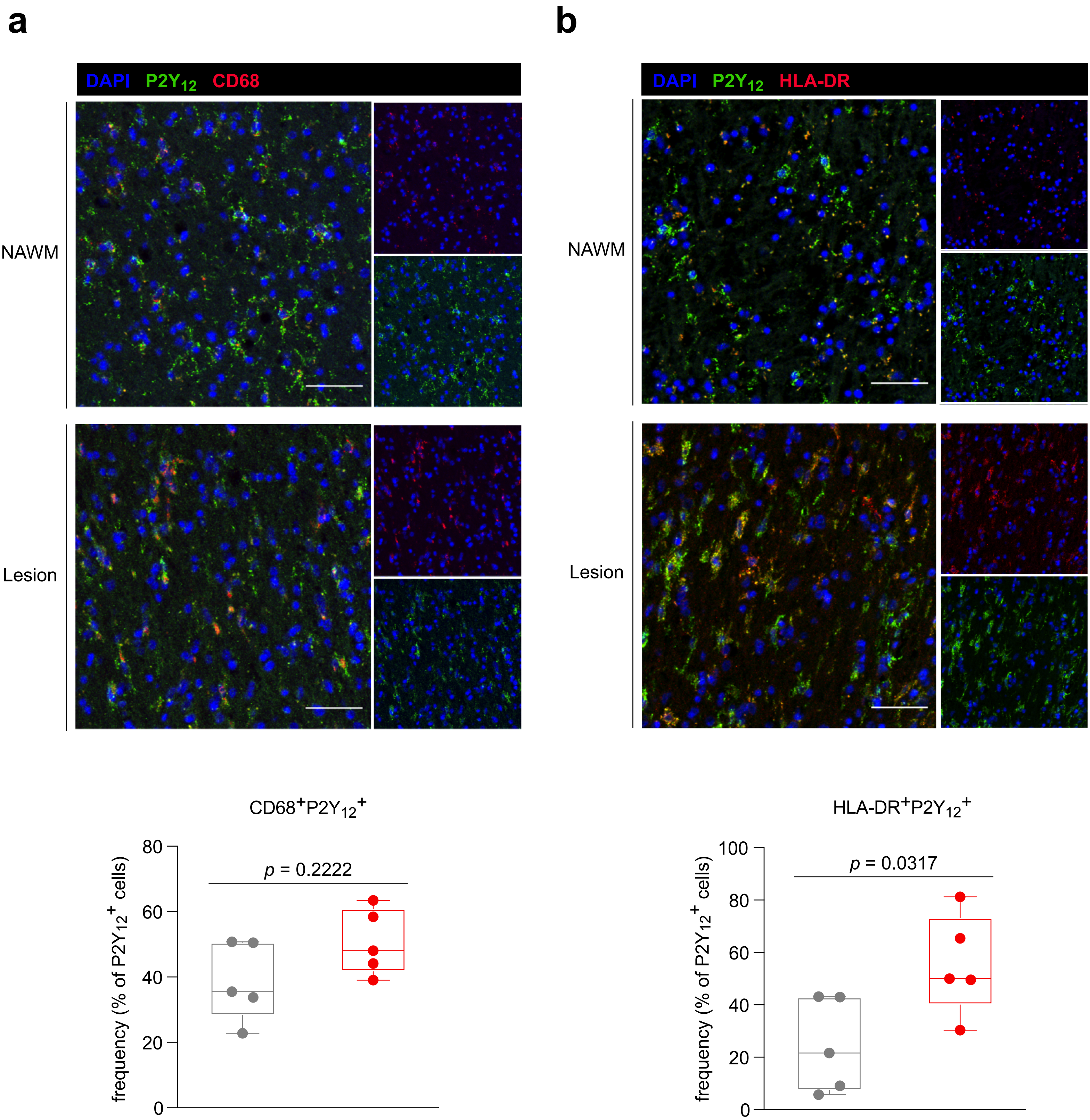

Supplementary Figure 3

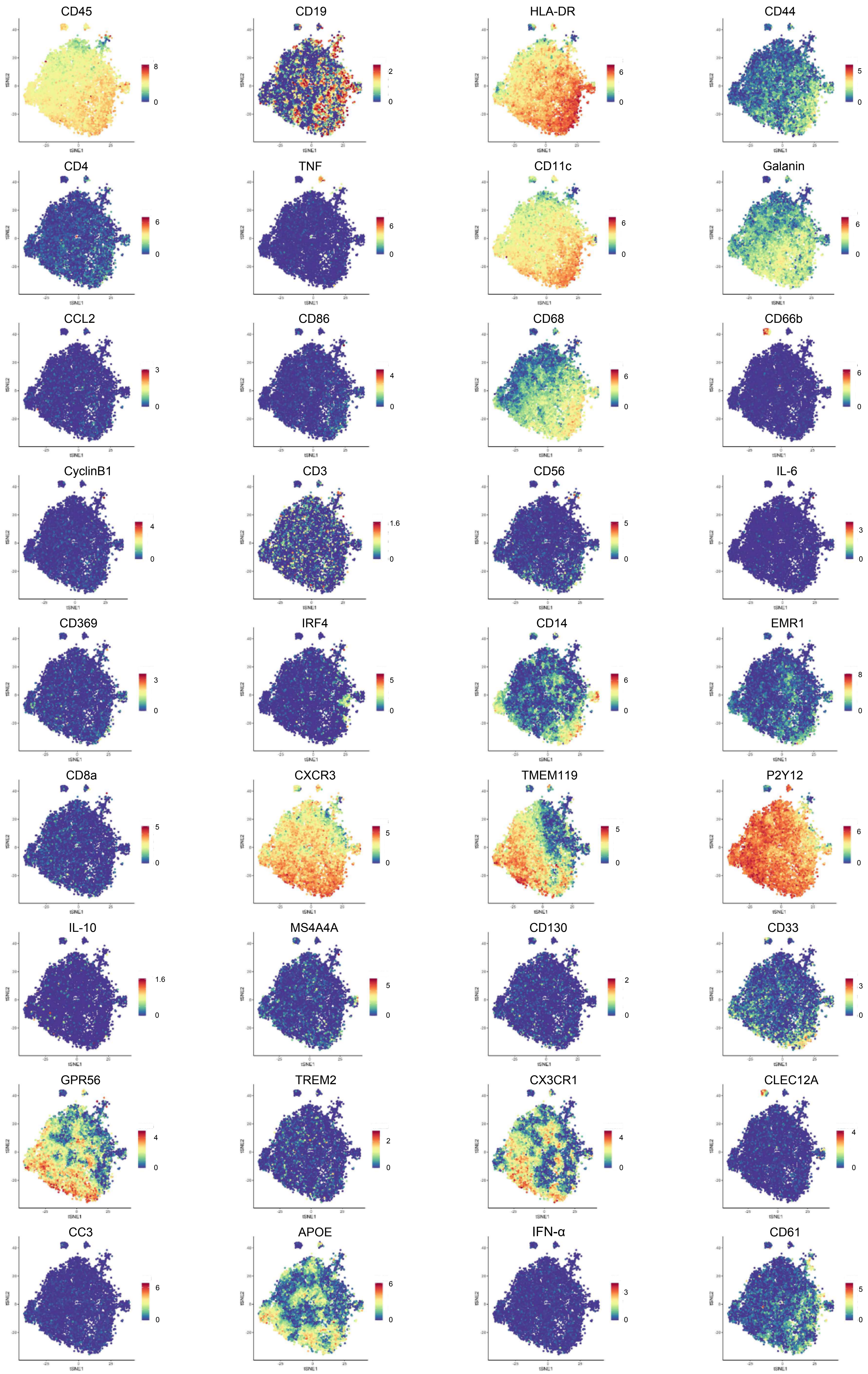

Supplementary Figure 4.

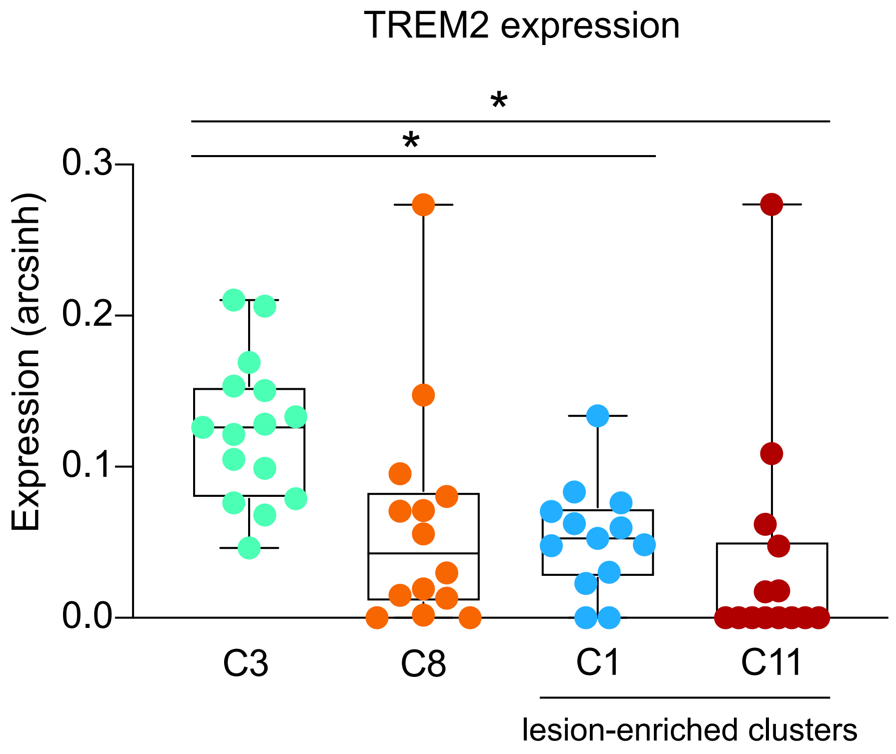

Supplementary Figure 5

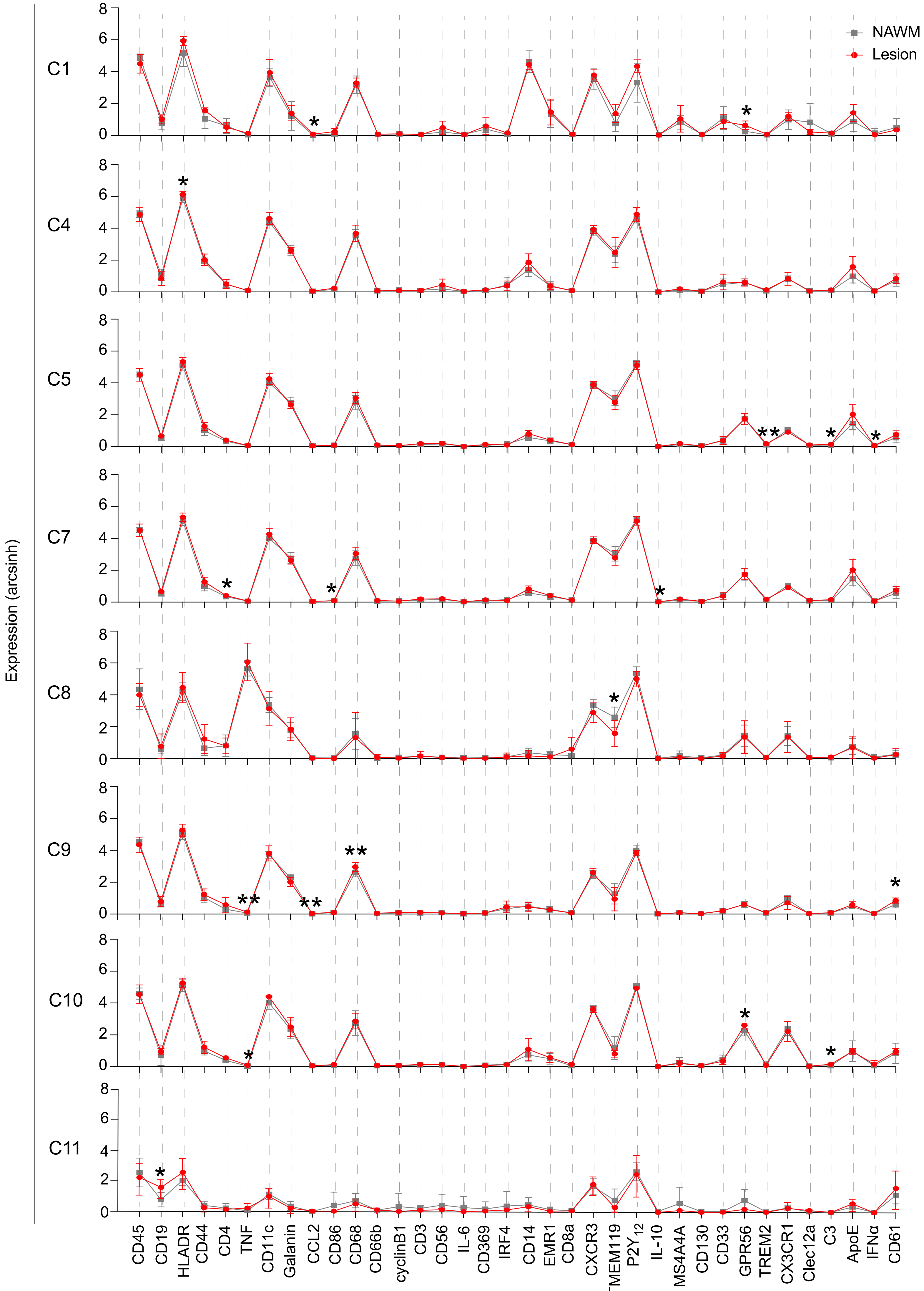

Supplementary Figure 6

a

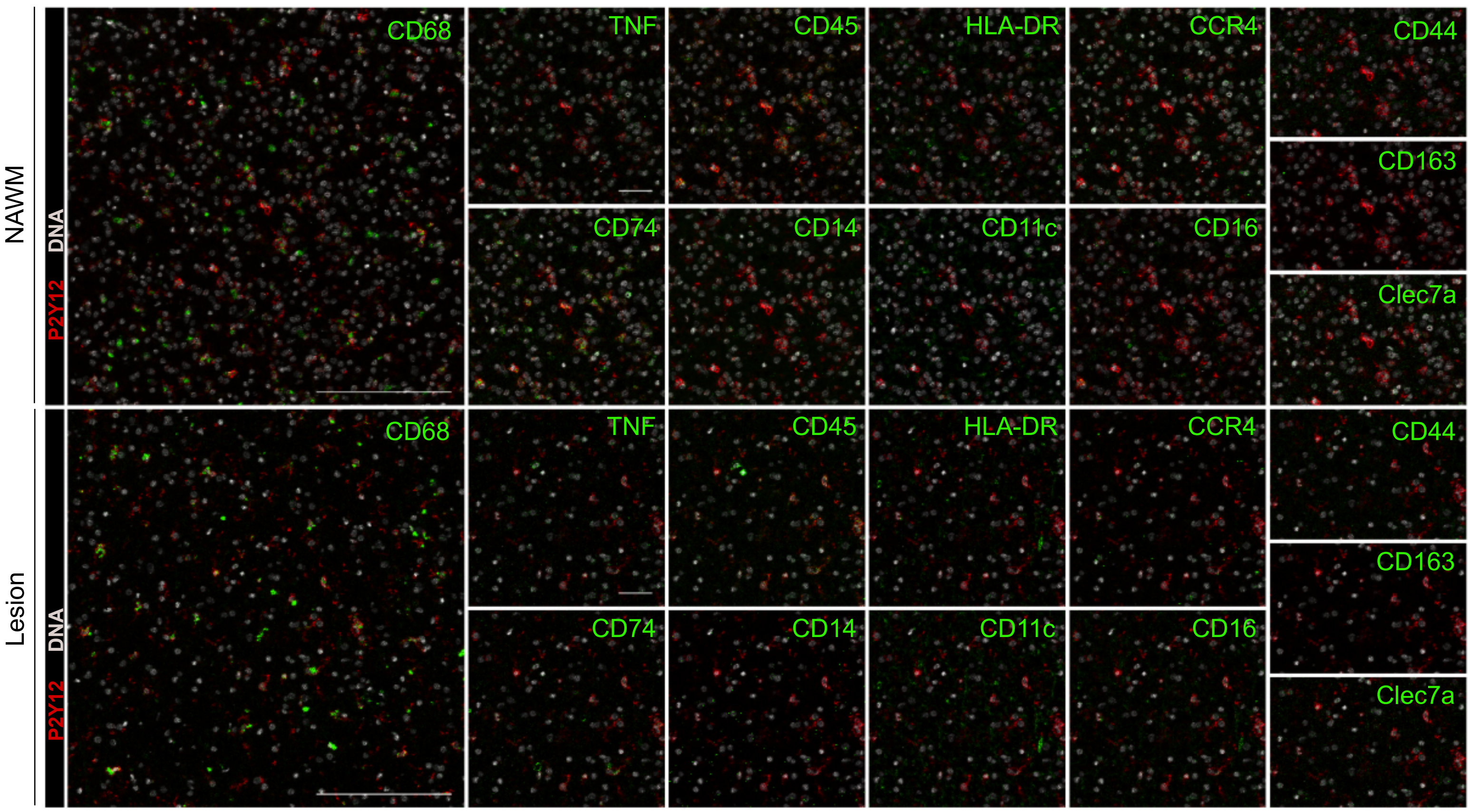

b

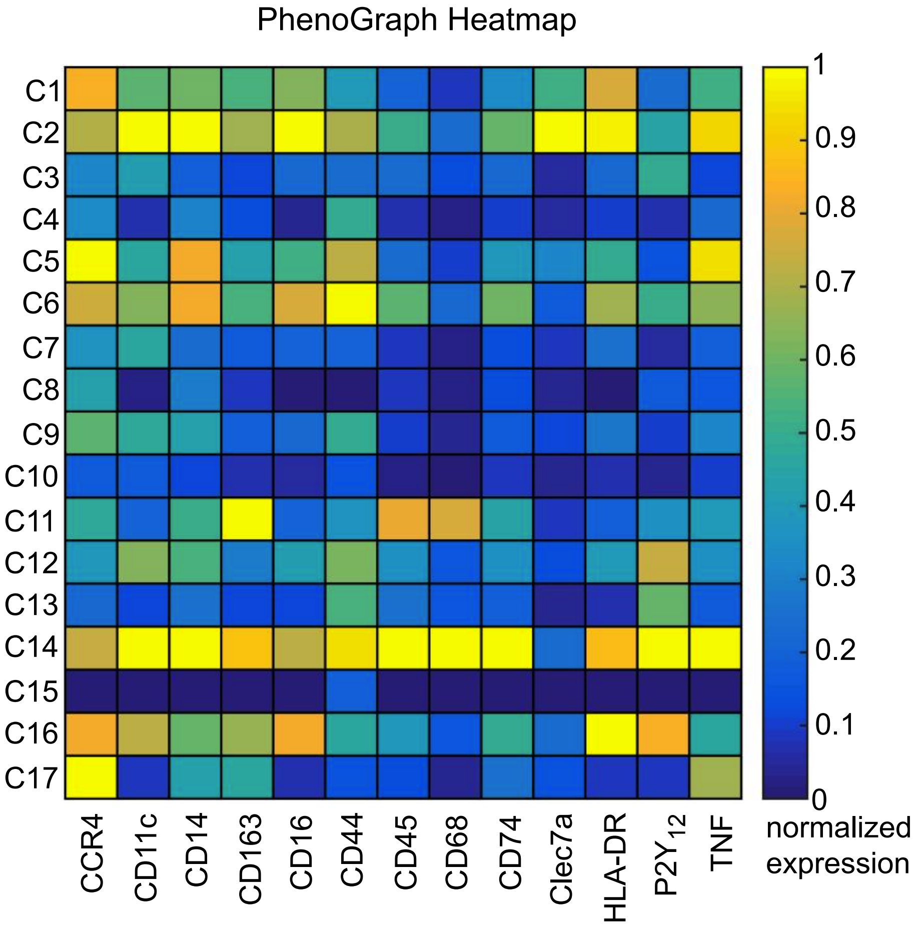

Supplementary Figure 7

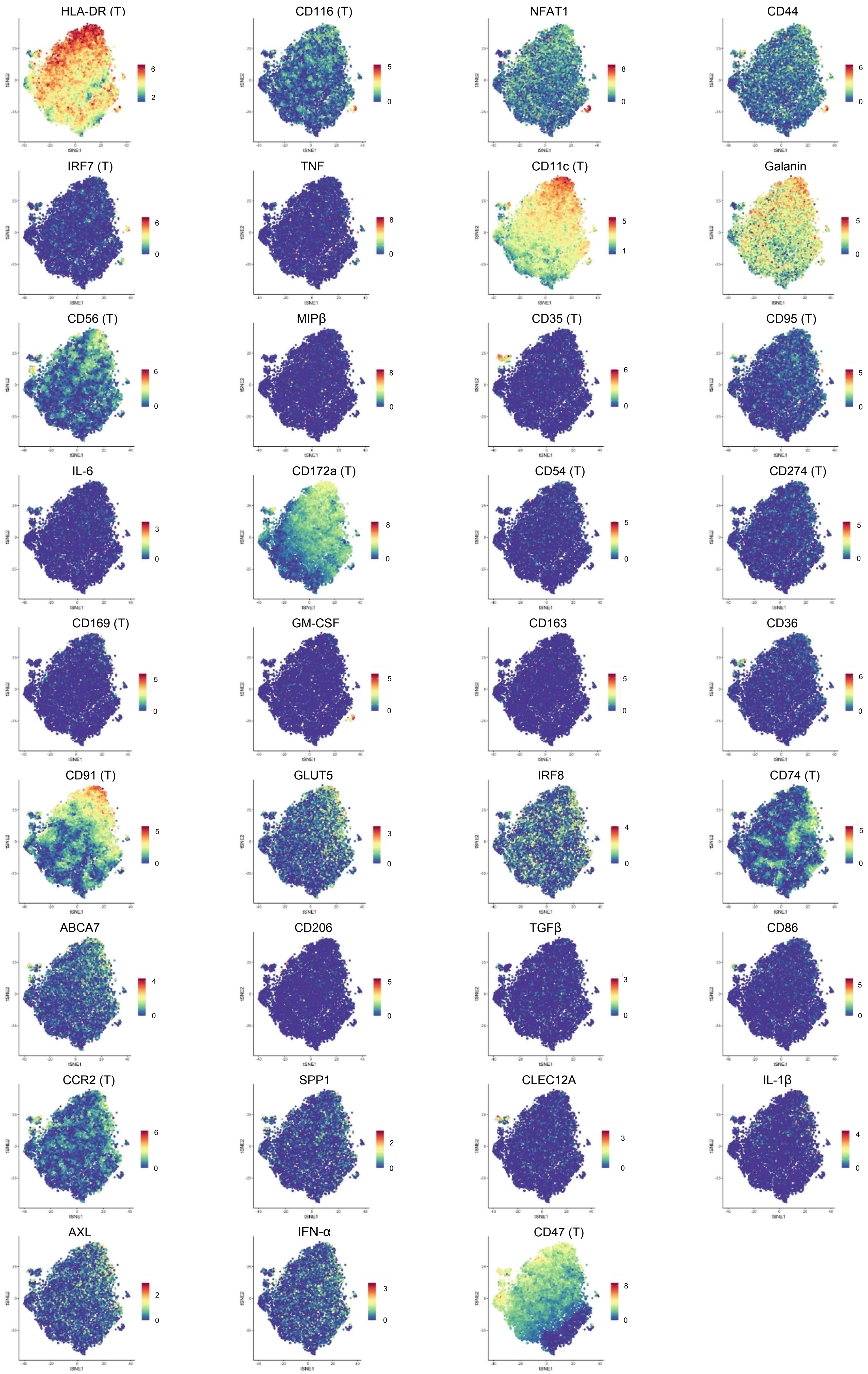

Supplementary Figure 8.

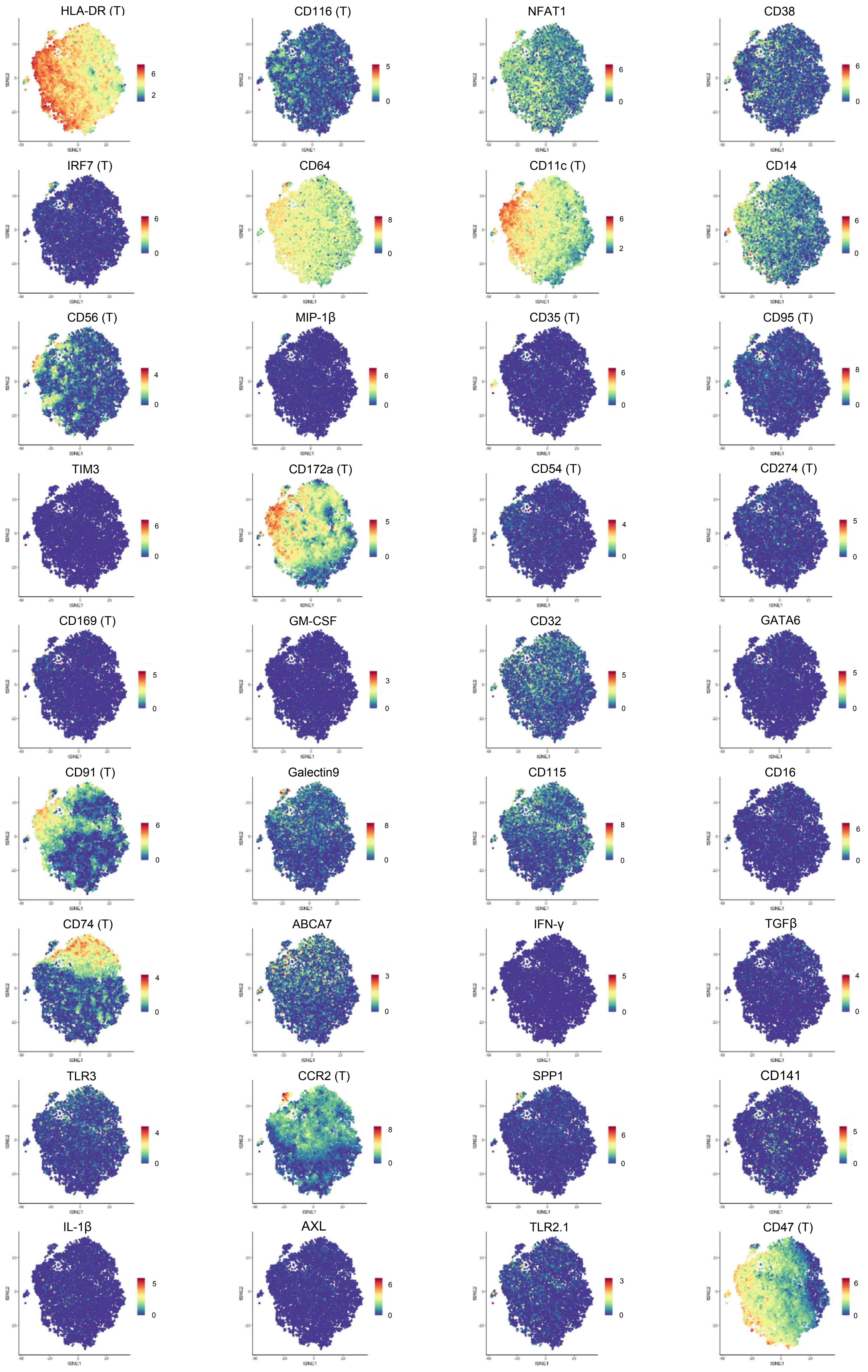

Supplementary Figure 9.

a

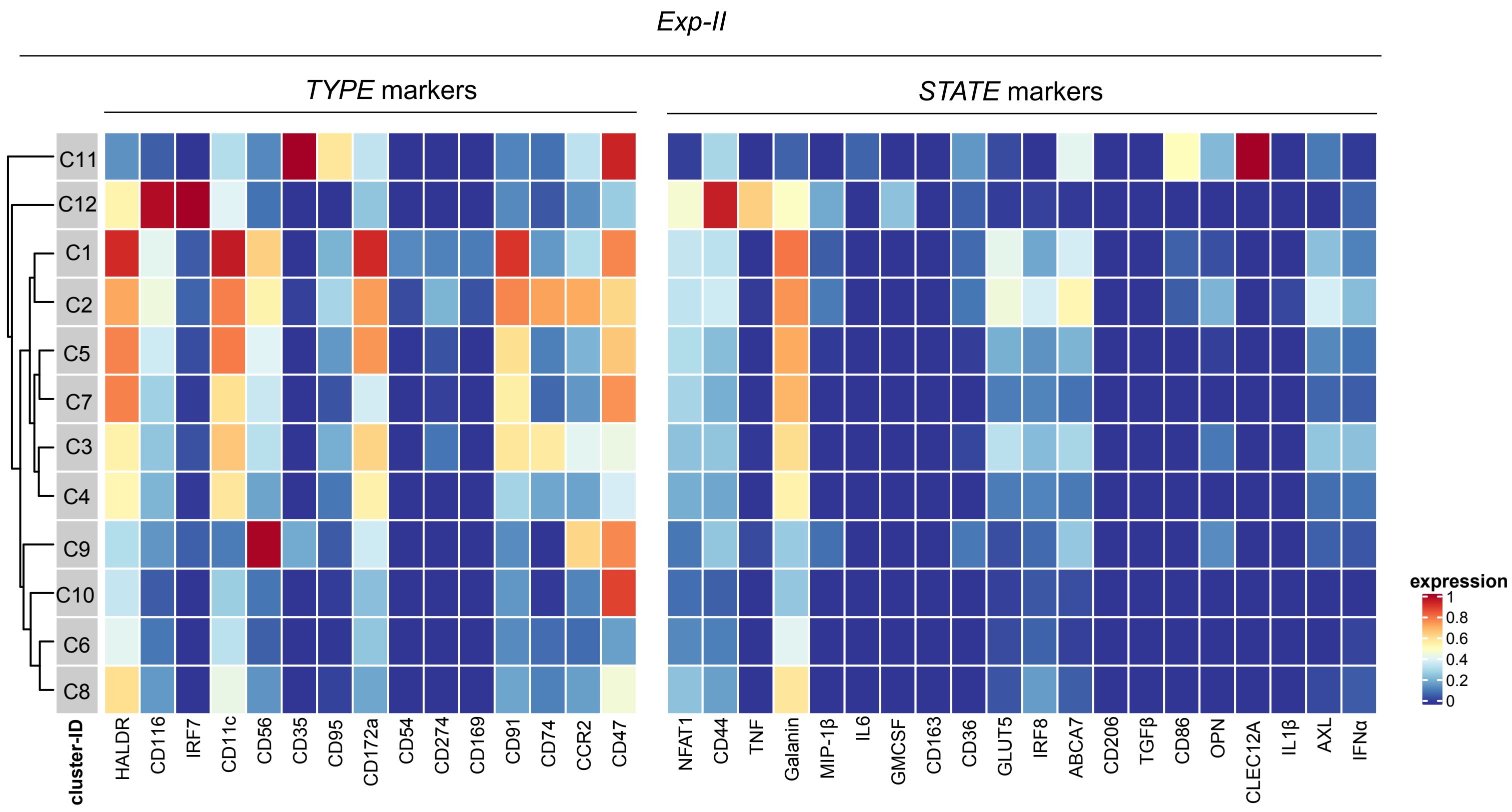

b

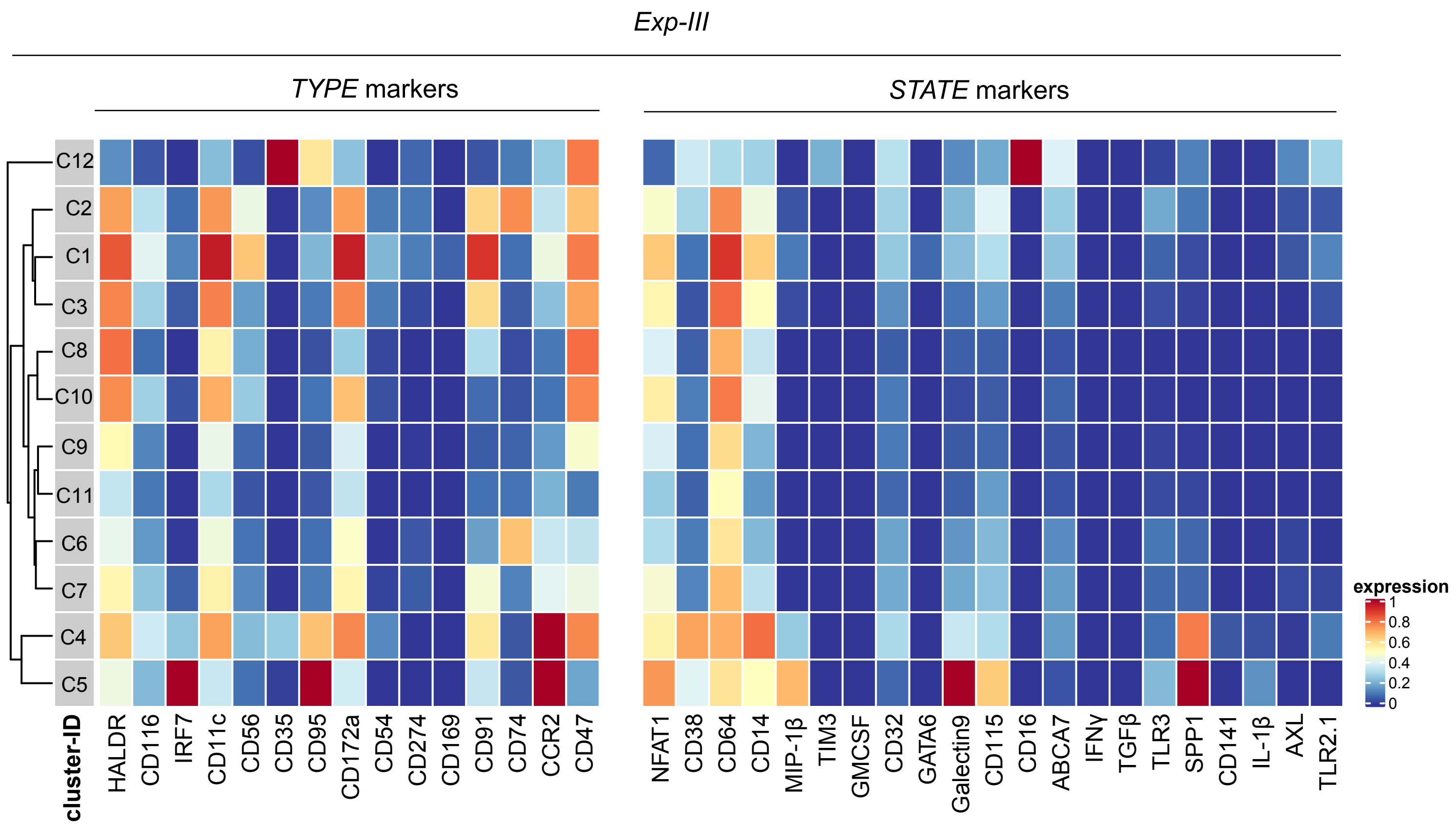

Supplementary Figure 10

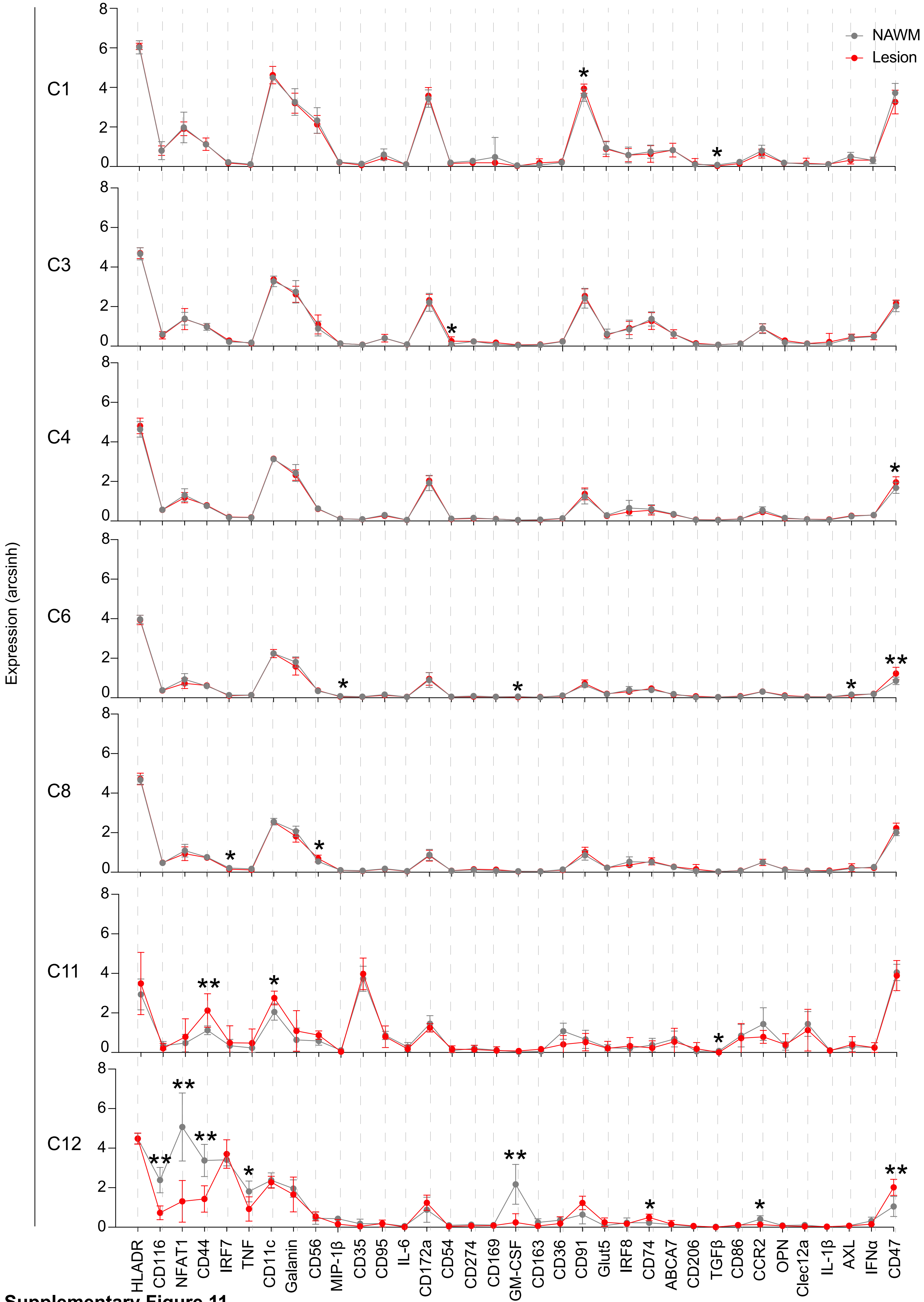

Supplementary Figure 11

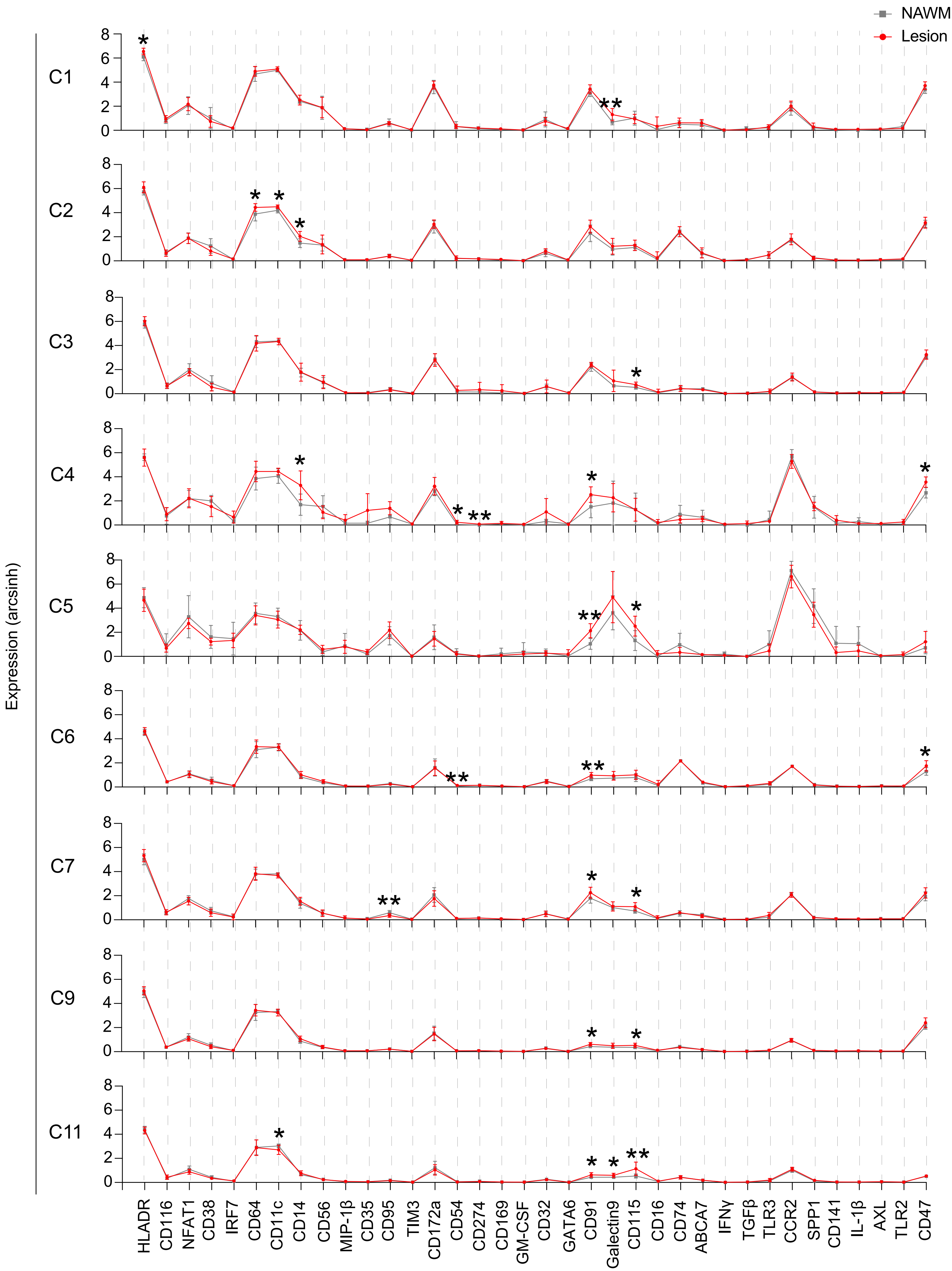

Supplementary Figure 12

Figure legends

Supplementary Figure 1 Flow cytometry analysis of microglia isolated from MS brain donors. Representative dot plots showing about 50% of total events were cells (SSC-A *vs* FSC-A) of which more than 90% are cell-singlets (FSC-W *vs* FSC-H and SSC-W *vs* SSC-H). Of note, these cell-singlets were identified as DNA ($^{191/193}$Ir)-positive in CyTOF, whereas the other events were DNA-negative. More than 70% of detected single cells were viable (Live/Dead *vs* FCS-A) and > 95% are myeloid cells ($CD45^+CD3^-CD19^-$ and $CD45^+CD11b^+$).

Supplementary Figure 2 MS lesion characterization. (**a**) Representative brain sections of control white matter (CON, upper image; scale bar = 1 mm) and normal appearing white matter (NAWM) as well as lesion-enriched white matter (Lesion) of donors with MS pathology (lower image; scale bar = 1 mm). NAWM tissue shows ramified microglia and intact myelin, based on HLA-DR and PLP staining, respectively. In active lesion, demyelinated center and active microglia/macrophages could be detected throughout the whole lesion. The majority of HLA-DR^+ cells in lesion MS are foamy microglia/macrophages. Scale bar of high resolution pictures is 50 µm. (**b**) Isolated $IRF8^+$ nuclei, enriched for microglia genes show conserved expression of homeostatic genes *CX3CR1*, *TMEM119, P2RY12* and *ADGRG1* in active MS lesions (n=5) as compared to NAWM (n=7), detected by RT-qPCR. (**c**) Microglial expression of CX3CR1, $P2Y_{12}$ and GPR56 is also not changed at protein level in active MS lesions (n=4-8) compared to NAWM (n=4-8), detected by flow cytometry. Statistical testing: Mann Whitney test and Wilcoxon paired *t-test*. (**d**) Representative laser confocal microscopic images of CON, NAWM and lesion WM tissues showing microglia as $P2Y_{12}^+$ cells (green), and nuclei are counterstained with DAPI (blue). The graph shows the quantification of $P2Y_{12}^+$ cells in CON, NAWM and lesion WM tissues. Statistical testing: Brown-Forsythe and Welch ANOVA test; * $p < 0.05$ and ** $p < 0.01$.

Supplementary Figure 3 Immunohistochemistry analysis of $P2Y_{12}^+$ cells. Representative laser confocal microscopic images of NAWM and lesion WM tissues showing CD68 (red, **a**) and HLA-DR (red, **b**) expression on $P2Y_{12}^+$ (green) microglia, and nuclei are counterstained with DAPI (blue). The graph shows the quantification of $CD68^+P2Y_{12}^+$ (a) and HLA-$DR^+P2Y_{12}^+$ (**b**) cells in NAWM and lesion WM tissues. Statistical testing: Mann-Whitney test; $p < 0.05$ was considered as statistically significant.

Supplementary Figure 4 Marker expression-*Exp-I*. The overlaid t-SNE plot of 8 NAWM and 7 lesion white matter samples. The 2D t-SNE maps were generated based on expression levels of all markers of *Exp-I* (Supplementary Table 8), each dot represents a cell and the color denotes the expression level of each targeted protein.

Supplementary Figure 5 TREM2 expression. Dot plot graph show TREM2 expression (*Exp-I*) of hoMG **C3** cluster compared to the lower abundant **C8** and enriched clusters **C1** and **C11** in active lesions.

Supplementary Figure 6 Cluster-wise assessment of phenotypic differences (*Exp-I*). The graphs showed differential marker expression (mean ± sd) between myeloid cells from NAWM and lesion WM tissues within each defined cluster. Small phenotypic differences between the studied groups were found in defined cluster **C1**, **C4**, **C5**, **C7**, **C8**, **C9**, **C10** and **C11**, whereas no significant differences in marker expression were observed in **C2**, **C3**, **C6**, and **C12**. Statistical testing: Mann-Whitney test; * $p < 0.05$ and ** $p < 0.05$.

Supplementary Figure 7 Analysis of myeloid cell heterogeneity using imaging CyTOF. (**a**) Representative IMC images of normal-appearing white matter (NAWM) and Lesion WM

(scale bar = 200 μm (low resolution) and 50 μm (high resolution)) show expression of 13 markers analyzed. (**b**) Heat map demonstrates the phenotypic diversity of all clusters characterized using PhenoGraph. The heat color shows expression levels of all markers used for the analysis.

Supplementary Figure 8 Marker expression-*Exp-II*. The overlaid t-SNE plot of 10 NAWM and 8 lesion white matter samples. The 2D t-SNE maps were generated based on expression levels of all markers of *Exp-II* (Supplementary Table 8), each dot represents a cell and the color denotes the expression level of each targeted protein.

Supplementary Figure 9 Marker expression-*Exp-III*. The overlaid t-SNE plot of 8 NAWM and 9 lesion white matter samples. The 2D t-SNE maps were generated based on expression levels of all markers of *Exp-III* (Supplementary Table 8), each dot represents a cell and the color denotes the expression level of each targeted protein.

Supplementary Figure 10 FlowSOM phenotypic heat maps – *Exp-II* and *-III*. Heat map cluster demonstrates the expression levels of all markers used for the cluster analysis in *Exp-II* (**a**) and *Exp-III* (**b**). Heat colors of expression levels have been scaled for each marker individually (to the 1st and 5th quintiles) (red, high expression; blue, low expression).

Supplementary Figure 11 Cluster-wise assessment of phenotypic differences (*Exp-II*). The graphs showed differential marker expression (mean ± sd) between myeloid cells from NAWM and lesion WM tissues within each defined cluster. Small phenotypic differences between the studied groups were found in cluster **C1**, **C3**, **C4**, **C6**, **C8** and **C11**, whereas strongly decreased expressions of CD116, NFAT1, CD44 and GM-CSF in lesion-associated myeloid cells were detected in cluster **C12**. No significant differences in marker expression

were observed in **C2**, **C5**, **C7**, **C9** and **C10**. Statistical testing: Mann-Whitney test; * $p < 0.05$ and ** $p < 0.05$.

Supplementary Figure 12 Cluster-wise assessment of phenotypic differences (*Exp-III*). The graphs showed differential marker expression (mean ± sd) between myeloid cells from NAWM and lesion WM tissues within each defined cluster. Small phenotypic differences between the studied groups were found in cluster **C1**, **C2**, **C3**, **C4**, **C5**, **C6**, **C7**, **C9** and **C11**, whereas no significant differences in marker expression were observed in **C8**, **C10** and **C12**. Statistical testing: Mann-Whitney test; * $p < 0.05$ and ** $p < 0.05$.
